# Local and distant tumor dormancy during early stage breast cancer are associated with the predominance of infiltrating T effector subsets

**DOI:** 10.1186/s13058-020-01357-9

**Published:** 2020-10-28

**Authors:** Hussein F. Aqbi, Cara Coleman, Melika Zarei, Saeed H. Manjili, Laura Graham, Jennifer Koblinski, Chunquing Guo, Yibin Xie, Georgi Guruli, Harry D. Bear, Michael O. Idowu, Mehran Habibi, Xiang-Yang Wang, Masoud H. Manjili

**Affiliations:** 1 Department of Microbiology & Immunology, VCU School of Medicine, Richmond, VA USA; 2 VCU Massey Cancer Center, 401 College Street, Richmond, VA 23298 USA; 3 College of Science, Mustansiriyah University, Baghdad, Iraq; 4 Emory University School of Medicine, Atlanta, GA USA; 5 Department of Biomedical Engineering, VCU School of Engineering, Richmond, VA USA; 6 Department of Surgery, VCU School of Medicine, Richmond, VA USA; 7 Department of Pathology, VCU School of Medicine, Richmond, VA USA; 8 Department of Human & Molecular Genetics, VCU School of Medicine, Richmond, VA USA; 9 VCU Institute of Molecular Medicine, Richmond, VA USA; 10 Peking Union Medical College, Beijing, China; 11 Department of Internal Medicine, VCU School of Medicine, Richmond, VA USA; 12 Department of Surgery, Johns Hopkins School of Medicine, Baltimore, MD USA

**Keywords:** Breast cancer, Tumor dormancy, T cells, Cancer immunotherapy, FVBN202 transgenic mouse

## Abstract

**Background:**

Although breast cancer mortality is a result of distant recurrences associated with the establishment of tumor dormancy, current clinical practice guidelines recommend a wait and watch approach for tumor recurrences. This is because of our limited understanding of tumor dormancy and insufficient evidence in support of immunological control of tumor dormancy.

**Methods:**

We used FVBN202 transgenic mice expressing rat neu oncogene in the mammary glands, and their parental FVB strain lacking neu expression. These models allowed the detection of tumor dormancy at distant sites using the rat neu protein as a tumor marker. We also used Ki67 for the detection of the indolent and quiescent types of tumor dormancy. Multicolor flow cytometry was used to detect dormant tumor cells and T cell subsets. Co-culture studies were performed to determine the role of T cells in preventing regrowth of dormant cells.

**Results:**

We demonstrated that dormant tumor cells were present at the site of primary breast cancer and at distant sites in the lungs and in the liver very early in the course of early stage breast cancer when no distant metastasis was evident. Dormant tumor cells were characterized as neu expressing Ki67^−^ and Ki67^low^ fractions associated with the induction of local immune responses predominated by CD4+ and CD8+ T effector cell subsets. The presence of neu-autoreactive T cells from FVBN202 mice only prevented regrowth of dormant cells. On the other hand, presence of neu-alloreactive anti-tumor T cells in FVB mice prior to tumor challenge resulted in the protection of mice from the dissemination of dormant tumor cells to distant organs.

**Conclusion:**

Our results suggest that immunotherapeutic targeting of semi-allogeneic mutant neoantigens during tumor dormancy might prevent distant recurrence of the disease.

## Background

Progress in cancer therapies has resulted in improved survival of patients with early stage breast cancer. However, mortality remains high in patients with distant recurrence of the disease after initially successful treatment of early stage breast cancer. Recent advances in immunotherapy of cancer by means of antibody therapies [1, 2] and immune checkpoint blockade [3–6] have prolonged survival of cancer patients, but many patients do not respond to these therapies [5, 7, 8] and those who respond would remain at risk of tumor recurrence [6, 9, 10]. Therefore, a relapse-free cancer cure remains a major challenge for cancer therapeutics. To this end, tumor recurrence has been attributed to the presence of dormant tumor cells in breast cancer patients [11, 12]. Reviews of the literature on cancer dormancy suggest that early stage primary cancers and distant recurrences of cancer as advanced stage diseases arise from naturally occurring tumor dormancy and treatment-induced tumor dormancy, respectively [13–17]. Among heterogeneous tumor cell population within primary breast cancer, quiescent tumor cells exist in the form of cellular dormancy. These quiescent cells are refractory to cancer therapies and become enriched following cancer therapies [18]. However, they could be susceptible to immunotherapy if targeted before clinical recurrences [19]. Recently, we have reported that dormant tumor cells established by chemotherapy or radiation therapy became resistant to additional doses of these therapies but remained susceptible to immunotherapy [20, 21]. Chemotherapy-induced immunogenic tumor dormancy maintained by the immune response has also been reported by others [22]. Although the equilibrium phase of tumor immunoediting has been suggested to be responsible for tumor dormancy [13, 23–25], there is no preclinical evidence demonstrating that cellular tumor dormancy or tumor relapse is associated with predominance of certain subsets of infiltrating T cells at the site of tumor dormancy. Here, we used the FVBN202 transgenic mouse model of neu-overexpressing breast cancer and the FVB parental strain to investigate the state of local and distant tumor dormancy during early stage breast cancer, as well as the role of the immune response or chemotherapy in controlling tumor dormancy and preventing tumor relapse. While FVBN202 transgenic mice tolerate neu-overexpressing mammary tumor similar to human counterpart, FVB mice mount alloreactive anti-neu immune responses and serve as negative control for studying distant tumor dormancy in FVBN202 transgenic mice. In the FVBN202 mice, we showed that local or distant tumor dormancy in the lungs or in the liver occurs very early during primary breast cancer when no metastatic cancer is evident, regardless of cancer therapies. Also, in the FVB mice that reject the tumor because of harboring pre-existing alloreactive T cells against rat neu antigen, we showed that presence of pre-existing anti-tumor T cells could completely eliminate primary tumor and prevent distant tumor dormancy. Our data suggests that a timely targeting of dormant tumor cells by an effective immunotherapy could prevent distant recurrence of the disease as an advanced stage breast cancer.

## Methods

### Animals

FVBN202 transgenic female mice or wild type FVB female mice (Jackson Laboratory; Bar Harbor, ME) were used. FVBN202 transgenic mice have been generated from wild type parental FVB by introducing non-mutated, non-activated rat neu transgene in the mammary glands under the regulation of the mouse mammary tumor virus promoter (MMTV) [26]. Similar to HER2 overexpressing human breast cancer, FVBN202 transgenic mice develop atypical ductal hyperplasia (ADH) and ductal carcinoma in situ (DCIS) preceding spontaneous mammary carcinoma by 5–6 months of age [27–29]. FVB mice do not express rat neu oncogene, and their T cells reject rat neu-overexpressing mouse mammary carcinoma (MMC) cells because of immune alloreactivity against the rat neu antigen [30, 31]. These studies have been reviewed and approved by the Institutional Animal Care and Use Committee at Virginia Commonwealth University.

### Tumor cell lines

The rat neu-overexpressing mouse mammary carcinoma (MMC) cell line was established from spontaneous mammary tumors harvested from FVBN202 mice [30]. R-Ctrl tumor cell line was established from dormant tumor cells in the lungs of FVBN202 mice bearing primary mammary carcinoma. R-FAC tumor cell line was established from dormant tumor cells in the lungs of FVBN202 mice bearing primary mammary carcinoma following FAC chemotherapy [10 mg/kg 5-Fluorouracil (5-FU) + 3 mg/kg Adriamycin (ADR) + 10 mg/kg Cyclophosphamide (CYP) every day for 9 days i.p.] [32–34]. R-FAC/AIT and R-FAC/AIT L tumor cell lines were established from dormant tumor cells in the lungs and in the liver, respectively, of FVBN202 mice bearing primary mammary carcinoma and receiving FAC chemotherapy and immunotherapy. Tumor cells were maintained in RPMI-1640 supplemented with 10% fetal bovine serum (FBS), and cells were kept at 37 °C with 5% CO_2_. Cells were routinely passaged when needed by detaching adherent cells using trypsin-EDTA (0.25%; Life Technologies). For flow cytometry, tumor cells were detached using 10 mM EDTA solution (Quality Biologicals Inc., Gaithersburg, MD).

### Detection of tumor dormancy and establishment of dormant tumor cell lines from the lungs or liver of FVBN202 mice

Rat neu oncogene is specifically expressed in the mammary glands of FVBN202 transgenic mice but not in FVB mice. Therefore, tumor dormancy can be detected in distant organs of FVBN202 mice by the detection of rat neu protein on gated viable cells that do not express Ki67. To isolate and establish dormant tumor cell lines from distant organs, the lungs and liver were collected under aseptic conditions, minced, and digested in trypsin-EDTA overnight at 4 °C. The following day, the suspension was incubated at 37 °C for 30 min, followed by gentle tissue homogenization and filtered through a 70-μm mesh to create a cellular suspension. The cell suspension was then washed twice with RPMI-1640 supplemented with 10% FBS. Residual red blood cells were then lysed using ACK lysing buffer, followed by an additional wash with RPMI-1640 10% FBS. The cell suspension was then cultured with RPMI-1640 + 20% FBS for 2 weeks followed by culture in RMPI-1640 supplemented with 10% FBS.

### Chemotherapy and adoptive T cell therapy

Three days after tumor challenge, FVBN202 mice were treated with low dose metronomic FAC (10 mg/kg 5-FU + 3 mg/kg ADR + 10 mg/kg CYP every day for 9 days i.p.) [32–34]. Reprogramming of tumor-sensitized autologous immune cells was performed as previously described by our group [20]. FVBN202 mice were inoculated with 3 million MMC in the mammary region, and growth was monitored by digital caliper. Spleens were harvested when tumor had reached ≥ 1000 mm^3^. Splenocytes were then cultured in complete medium (RPMI 1640 supplemented with 10% FBS, l-glutamine (2 mM), 100 U/ml Penicillin, and 100 μg/ml Streptomycin) and were stimulated with Bryostatin 1 (2 nM) (Sigma, Saint Louis, MO), Ionomycin (1 μM) (Calbiochem, San Diego, CA) (B/I), and 80 U/ml of IL-2 (Peprotech) for 16–18 h. Lymphocytes were then washed three times and cultured in complete medium with recombinant murine IL-7 and IL-15 (20 ng/ml of each, Peprotech). After 24 h, 20 U/ml of IL-2 was added to the culture and the following day cells were washed and cultured with 40 U/ml of IL-2. After 48 h, cells were washed again and 40 U/ml of fresh IL-2 was added. Twenty-four hours later, lymphocytes were washed again and cultured with 40 U/ml of fresh IL-2. Lymphocytes were harvested 24 h later on the 6th day and were used for in vitro studies (cytotoxicity experiments) or in vivo studies of adoptive T cell therapy (AIT). FVBN202 mice were injected i.p. with CYP (100 mg/kg) to induce lymphopenia 1 day prior to AIT. AIT was performed i.v. at a dose of 70 × 10^6^ cells/mouse 3 days after tumor challenge or once the tumor became palpable (50–70 mm^3^). Mice received i.p. injections with 100 μg of anti-programmed cell death protein 1 (anti-PD-1) antibody (Bioxcell) in a total volume of 300 μl of dilution buffer (5 times every 3 days) [35]. As soon as the tumor reached 1000 mm^3^ in the control group (primary tumor model) or when they had a weight loss of ≥ 10% (experimental metastatic model where animals were injected with 1 million tumor cells/mouse i.v.), animals were sacrificed and their tumors, lungs, and liver were collected in complete medium for further analysis.

### Flow cytometry

Multicolor staining and flow cytometry analyses of the tumor, lungs, or liver were performed as previously described by our group [20], with some modifications. Infiltrating lymphocytes or neu-positive tumor cells were analyzed by multicolor staining of resected tissues. The tissues were minced into smaller pieces using scissors and forceps. A plunger from a disposable 5-ml syringe was used to mechanically disaggregate the tissues against a secured mesh filter. Complete medium was used to wash any remaining cells through the filter. Ki67 expression was determined as previously described by our group [20, 21]. Cells were fixed with 70% ethanol and stained with anti-Ki67 for 30 min at room temperature. Prior to fixation, cells were stained with Fixable Viability Stain (FVS) for 20 min at room temperature, washed, and stained with anti-neu antibody for 20 min on ice. Cells were washed twice with FACS buffer (1× PBS, 10% FBS, 0.1% sodium azide) prior to data acquisition. Multicolor data acquisition was performed using a LSRFortessa X-20 (BD Biosciences) and an ImageStreamX Mark II Imaging Flow Cytometer (Millipore Sigma, Billerica, MA). Data are analyzed using FCS Express v5.0 (De Novo Software; Glendale, CA). Reagents used for flow cytometry were as follows: anti-CD16/32 Ab (Fc blocker), FITC-anti-CD4, APC-anti-mouse IgG2a, BV421-anti-mouse IgG, BV711-anti-CD8, PE-anti-CD8, FITC-anti-CD3, PE-anti-Ki67, FITC-anti-CD45, BV785-anti-CD45, APC-anti-CD326, FITC-anti-CD326, BV421-anti-CD44, and APC-anti-CD62L, all of which were purchased from Biolegend (San Diego, CA). BD Horizon APC/Cy5-FVS and BUV395-anti-CD3 (SK7) were purchased from BD Biosciences (Franklin Lakes, NJ). Mouse anti-rat neu (anti-c-Erb2/c-Neu; 7.16.4) was purchased from Calbiochem (Billerica, MA, USA). All reagents were used at the manufacturer’s recommended concentration. The staining results are presented as percentage or mean fluorescence intensity (MFI). T cell subsets as T effector (Te, CD44+CD62L−), T effector/memory (Tem, CD44+CD62L^low^), T central/memory (Tcm, CD44+CD62L^high^), or T naïve (Tn, CD44−CD62L+) were analyzed. Cells with autofluorescence or FVS single color were used to gate for Ki67^+^ cells.

### Immunohistochemistry

Formalin-fixed, paraffin-embedded biopsy specimens and parallel surgical excisions from patients with early stage breast cancer were subjected to immunohistochemistry (IHC) staining using anti-human Ki67 antibody (Dako, clone 30-9). Results were analyzed using the Vectra® Polaris™ Automated Quantitative Pathology Imaging System and InForm software (PerkinElmer) (1 × 1 field, 20× resolution).

### Statistical analysis

Analyses were performed using one-tailed or two-tailed Student’s *t* test per the specific hypothesis. Three to five mice per group were used, and because of low variations within each group, a minimum of three mice per group was sufficient for tumor studies, as previously reported in these animal models [20, 21, 30]. Significances are shown as *< 0.05, **< 0.005, or ***< 0.0005.

### Study approval

Animal studies have been reviewed and approved by the Institutional Animal Care and Use Committee (IACUC) at Virginia Commonwealth University. Residual breast tumor surgical excisions were collected and used under Institutional Review Board (IRB) protocol# HM2471 of the Tissue and Data Acquisition and Analysis Core (TDAAC), the Biorepository core at Virginia Commonwealth University. All patients whose specimens are collected, banked, and used for research gave informed consent to participate in this research. The Biorepository core is also able to obtain consent from patients on behalf of the researcher for core biopsy prior to surgery for research use.

## Results

### Cancer therapies increase the proportion of Ki67^−^ dormant tumor cells at the site of primary mammary carcinoma

**Fig. 1:**
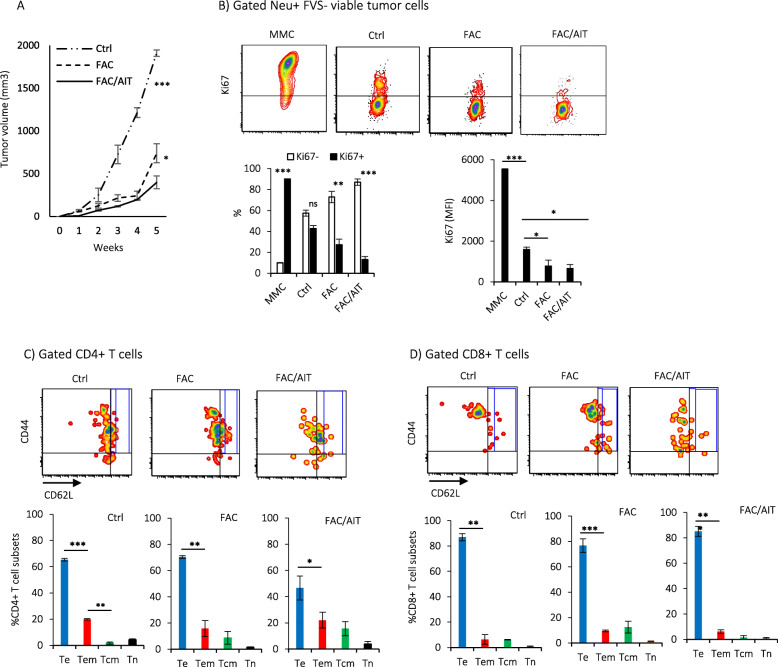
Local tumor dormancy before or after cancer therapies is associated with the predominance of infiltrating T effector cells. Female FVBN202 mice (8–10 weeks old) were challenged with neu-overexpressing MMC in the mammary region s.c. (3 × 10^6^ cells/mouse). Three days after challenge, tumor-bearing animals were split into three groups: one group received no treatment (Ctrl); second group received nine daily doses of FAC chemotherapy (10 mg/kg 5-FU + 3 mg/kg Adriamycin + 10 mg/kg Cyclophosphamide, i.p.) without immunotherapy (FAC) or followed by AIT and anti-PD-1 antibody therapy (FAC/AIT). Immunotherapy was started 1 week after the completion of FAC chemotherapy by a single i.v. injection of tumor-reactive immune cells (70 million cells/mouse) followed by five doses of anti-PD-1 antibody (100 mg/kg, every 3 days, i.p.). Animals were sacrificed 5 weeks after tumor challenge. **a** Tumor growth in the mammary region was measured using a digital caliper. **b** Expression of Ki67 on Fixable Viability Stain negative (FVS−) neu+ viable MMC cell line, or resected mammary tumors from the Ctrl, FAC, or FAC/AIT groups as percentage or mean fluorescence intensity (MFI). **c**, **d** Gated FVS− CD4+ or CD8+ T cells in resected mammary tumors were analyzed for T effector cells (Te, CD44+CD62L−), T effector/memory cells (Tem, CD44+CD62L^low^), T central/memory cells (Tcm, CD44+CD62L^high^), or T naïve cells (Tn, CD44−CD62L+) at the tumor site

**Fig. 2:**
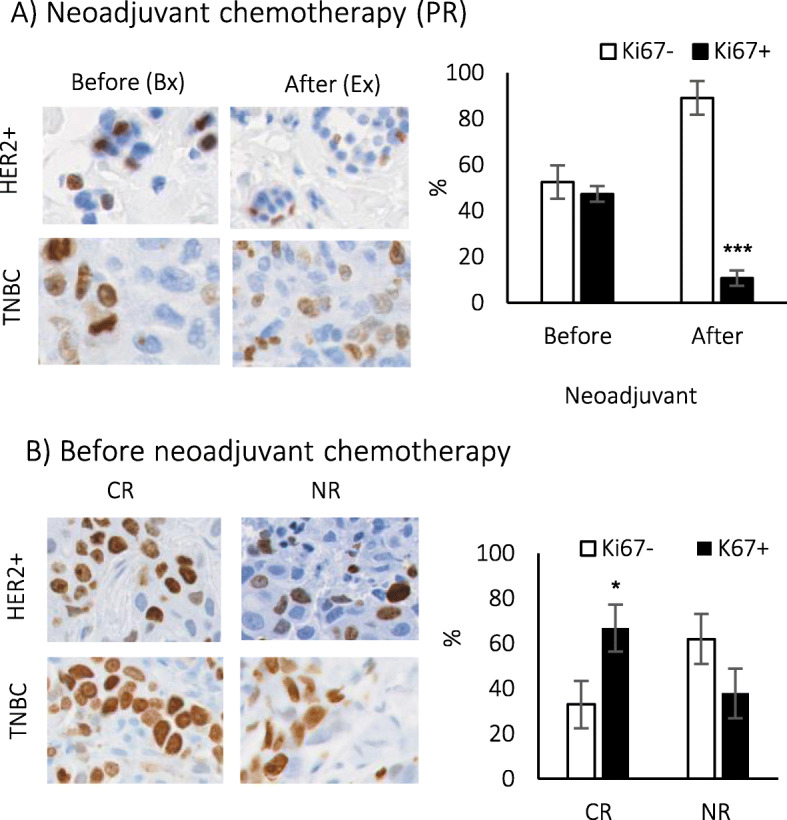
Neoadjuvant therapies induce local tumor dormancy in patients with early stage breast cancer. Patients with HER2-positive breast cancer (stages I–III) received neoadjuvant Pertuzumab, Trastuzumab, Docetaxel, and Carboplatin, and those with TNBC received neoadjuvant Taxol, Carboplatin, Adriamycin, and Cytoxan, followed by surgery. **a** Tumor biopsies (before) and surgical excisions (after), neoadjuvant therapies of patients with triple-negative breast cancer or HER2-positive breast cancer who partially responded (PR) to neoadjuvant therapies, were subjected to Ki67 staining. IHC analysis showing Ki67^+^ (brown) and Ki67^−^ (blue), and percent Ki67− or Ki67+ in tumor biopsies (before, *n* = 4) or surgical excisions (after, *n* = 4) were analyzed using the Vectra® Polaris™ Automated Quantitative Pathology Imaging System and InForm software (PerkinElmer) (1 × 1 field, 20× resolution). **b** Biopsy specimens of patients with early stage breast cancer who showed a complete response (CR) or no response (NR) to neoadjuvant therapies (5 patients per group) were analyzed for the expression of Ki67

We have reported the presence of slow-cycling Ki67^low^ and G0-arrested Ki67^−^ cells as indolent and quiescent types of tumor dormancy, respectively [20, 21]. While Ki67^−^ cells did not undergo cell division, slow-cycling Ki67^low^ showed sluggish cell proliferation counterbalanced by cell death [21]. Here, we wanted to determine whether cancer therapies select for Ki67^low^ indolent or Ki67^−^ quiescent dormant tumor cells. FVBN202 transgenic mice were challenged with MMC and received cancer therapies 3 days after tumor challenge. Control groups without cancer therapies (Ctrl) and naïve mice without tumor challenge were used as positive and negative controls, respectively. We used FAC chemotherapy without additional treatment (FAC) or followed by a single dose of AIT and 5 doses of anti-PD-1 therapy (FAC/AIT). Cancer therapies resulted in a significant inhibition of primary tumor growth compared with untreated control group (Fig. 1a). Compared to proliferating MMC tumor cell line showing a significant predominance of Ki67^+^ cells, tumor cells in control mice showed an increased Ki67^−^ fraction and lost the predominance of Ki67^+^ fraction with a significant drop in the intensity of Ki67 (Fig. 1b). Significant predominance of Ki67^−^ fraction became evident only after FAC or FAC/AIT cancer therapies (Fig. 1b). After cancer therapies, Ki67^+^ cells became slow-cycling Ki67^low^ as indicated by significant decreases in the intensity of Ki67 compared with control mice (Fig. 1b, MFI < 600). A loss of predominance of Ki67^+^ tumor cell fraction was associated with the predominance of tumor infiltrating CD4+ and CD8+ Te subsets in all animals with mammary carcinoma (Fig. 1c, d). A predominance of Te cells was associated with the expression of PD-1 on tumor infiltrating cells (Fig. S1A). In addition, the expression of PD-L1 on tumor cells was significantly increased in vivo, with the FAC/AIT group showing a highest increase in PD-L1 in their primary tumor (Fig. S1B). In order to determine clinical relevance of our findings in different breast cancer types, a pilot study was performed using tumor specimens from HER2-positive or triple-negative breast cancer patients with early stage (I–III) disease. Patients who partially responded to neoadjuvant therapies (PR) showed the presence of Ki67^−^ dormant cells (blue color) in the biopsies before neoadjuvant therapies (Bx, before) and surgical excisions after neoadjuvant therapies (Ex, after) (Fig. 2a). Ki67^−^ dormant cells were significantly enriched in surgical excisions after neoadjuvant therapies (Fig. 2a). Ki67^high^ tumor cells (dark brown) were present in biopsies, but they disappeared after neoadjuvant therapies, and almost all Ki67^+^ cells in surgical excisions were Ki67^low^ dormant cells (light brown) (Fig. 2a). Biopsy specimens (Bx) of patients who completely responded to neoadjuvant therapies (CR) contained a significantly higher proportion of Ki67^+^ to Ki67^−^ cells while non-responders (NR) had a proportionate number of Ki67^+^ and Ki67^−^ cells (Fig. 2b).

### Retention of distant tumor dormancy in the lungs or in the liver is associated with the predominance of infiltrating CD4+ and CD8+ Te subsets

**Fig. 3:**
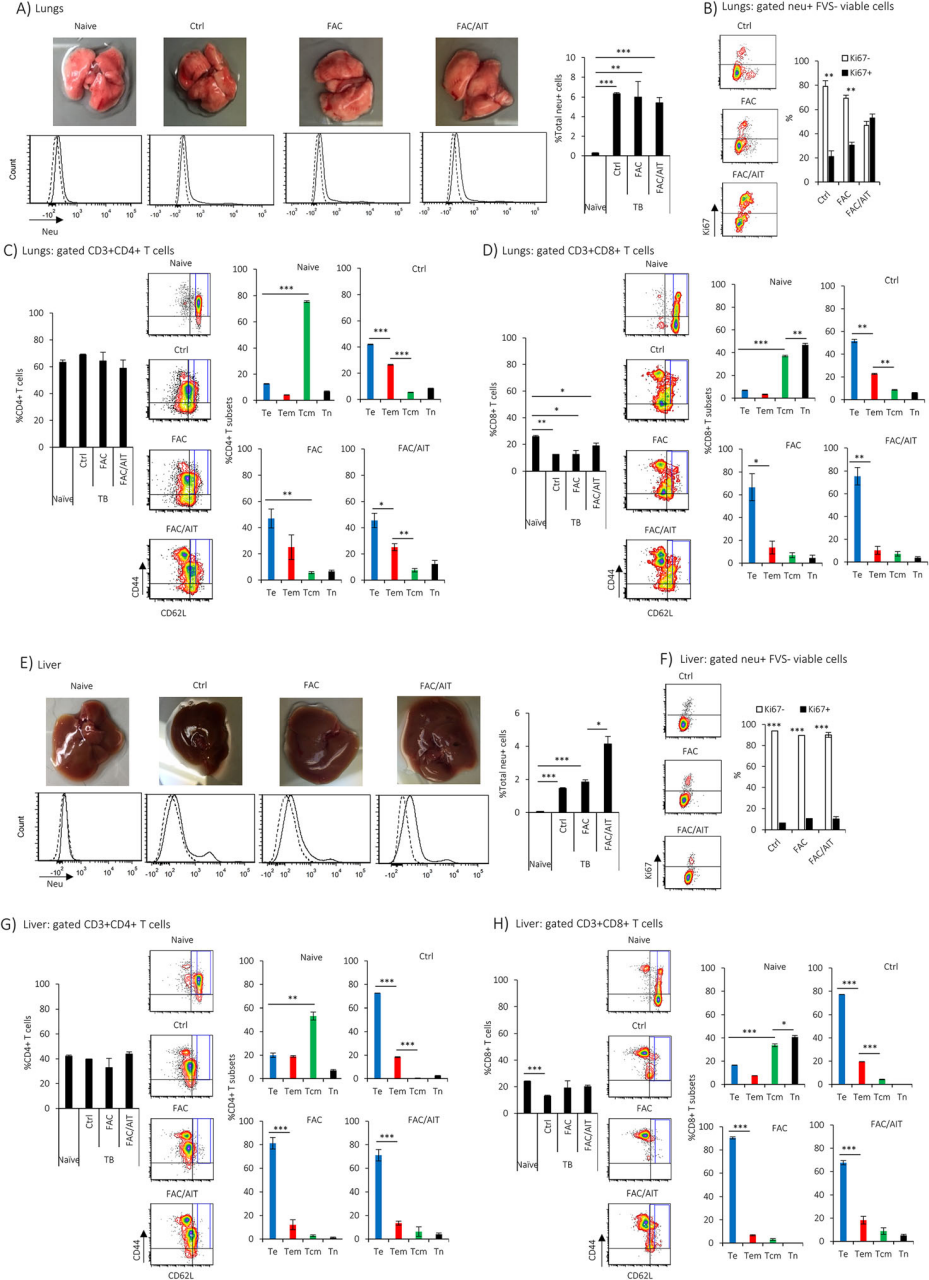
FVBN202 transgenic mice bearing early stage primary breast cancer show distant tumor dormancy regardless of cancer therapies. Female FVBN202 transgenic mice (8–10 weeks old) were challenged with neu-overexpressing MMC in the mammary region (3 × 10^6^ cells/mouse) or did not receive any tumor (naïve). Three days after challenge, tumor-bearing animals were split into three groups: one group received no treatment (Ctrl); second group received nine daily doses of FAC chemotherapy (10 mg/kg 5-FU + 3 mg/kg Adriamycin + 10 mg/kg Cyclophosphamide, i.p.) without immunotherapy (FAC) or followed by AIT and anti-PD-1 antibody therapy (FAC/AIT). Immunotherapy was started 1 week after the completion of FAC by a single i.v. injection of tumor-reactive immune cells (70 million cells/mouse) followed by five doses of anti-PD-1 antibody (100 mg/kg, every 3 days, i.p.). Animals were sacrificed 5 weeks after tumor challenge. Neu+ dormant tumor cells in the lungs (**a**) or in the liver (**e**) were detected on FVS negative (FVS−) viable cells (%total neu+ cells). Ki67 expression is shown on gated FVS− neu+ viable cells in the lungs (**b**) or in the liver (**f**). Gated FVS−CD3+ cells were analyzed for CD4+ or CD8+ T cells in the lungs (**c**, **d**) or in the liver (**g**, **h**). Gated FVS−CD3+CD4+ or CD8+ T cells were analyzed for T effector cells (Te, CD44+CD62L−), T effector/memory cells (Tem, CD44+CD62L^low^), T central/memory cells (Tcm, CD44+CD62L^high^), or T naïve cells (Tn, CD4−CD62L+) in the lungs (**c**, **d**) or in the liver (**g**, **h**)

In order to determine whether dormant tumor cells were disseminated in distant organs prior to visible tumor metastasis, the whole lungs and livers of FVBN202 transgenic mice were subjected to flow cytometry analysis before or after cancer therapies. FVBN202 transgenic mice being tumor-free (naïve) or bearing primary mammary carcinoma (TB) served as negative and positive controls for distant tumor dormancy, respectively. No visible tumor mass in the lungs or in the liver was detected (Fig. 3a, e, upper panel). In tumor-bearing, but not in naïve, mice, neu-positive viable dormant tumor cells were detected in the lungs (Fig. 3a, lower panel) or in the liver (Fig. 3e, lower panel). The neu-positive viable dormant tumor cells contained a predominant Ki67^−^ quiescent fraction in the lungs (Fig. 3b, Ctrl and FAC) or in the liver (Fig. 3f, all groups), except for the FAC/AIT group showing equal proportions of Ki67^−^ quiescent and Ki67^+^ indolent fractions only in the lungs (Fig. 3b). We have previously reported that maintenance of dormancy in Ki67^+/low^ indolent cells was through sluggish cell proliferation counterbalanced with cell death [21]. Equal proportions of Ki67^−^ quiescent and Ki67^+^ indolent fractions only in the lungs of the FAC/AIT group were associated with a significantly reduced expression of PD-1 on infiltrating T cells as well as a significantly increased expression of PD-L1 expression on dormant cells only in the lungs not in the liver (Fig. S2).

Analysis of viable CD4+ T cells in the lungs (Fig. 3c) or in the liver (Fig. 3g) showed shifts from predominant Tcm subset in naïve mice to predominant Te and Tem subsets in the control group, and a predominant Te subset in the FAC and FAC/AIT groups. Similarly, predominant CD8+ Tcm and Tn subsets in the lungs (Fig. 3d) or in the liver (Fig. 3h) of naïve mice were shifted to predominant Te and Tem subsets in the control group, and a predominant Te subset in the FAC and FAC/AIT groups. In order to determine whether the retention of tumor dormancy in distant organs was not merely because of the tissue microenvironment, FVBN202 mice were challenged with MMC via intravenous injection. Because of a systemic administration of MMC, animals developed progressive experimental metastasis in the lungs but not in the liver within 3–4 weeks after tumor challenge (Fig. S3). In a separate group of mice, primary tumors were resected, and animals were sacrificed 3 months after survival surgery showing no visible metastatic tumor or tumor lesions upon histological examination (Additional file 1).

### Dormant tumor cells isolated from the lungs can resume proliferation ex vivo

**Fig. 4:**
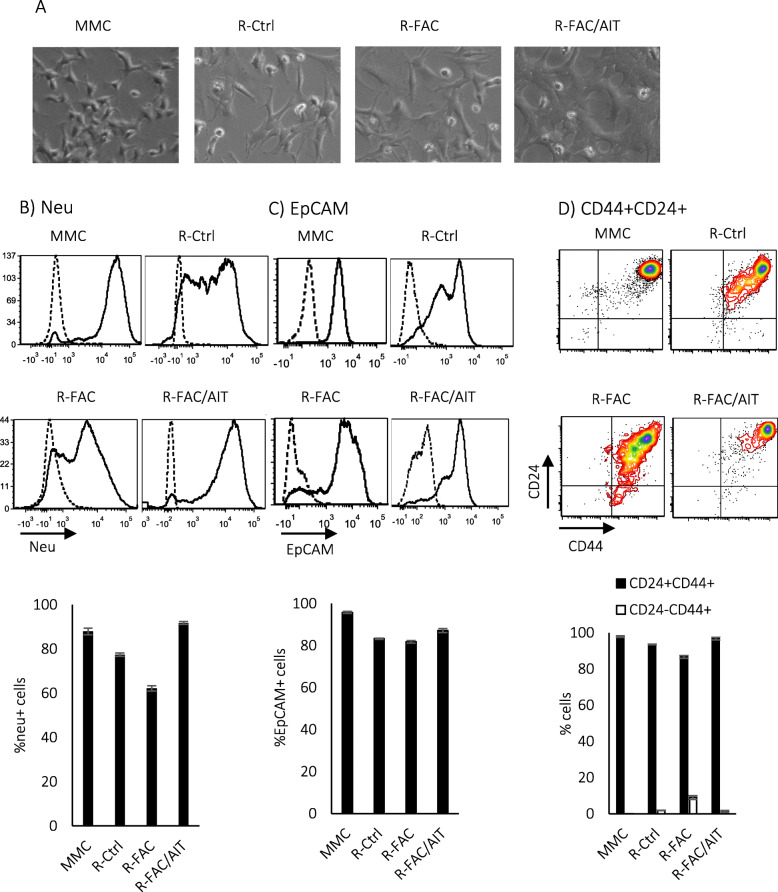
Dormant tumor cells can be recovered from distant organs, ex vivo. Lungs that did not have any visible tumor were obtained from FVBN202 transgenic mice bearing primary tumor in the mammary region who received no treatment (Ctrl), FAC chemotherapy (FAC), or FAC chemotherapy followed by AIT and anti-PD-1 antibody therapy (FAC/AIT). MMC tumor cell line was used as positive control for the expression of neu. **a** Representative pictures of MMC and dormant tumor cells recovered from the lungs. **b** Gated FVS− viable cells were analyzed for the detection of neu+ cells. **c** Gated FVS− neu+ viable cells were analyzed for the expression of EpCAM. **d** Gated FVS− viable cells were analyzed for the expression of CD44 and CD24. Data represents triplicates

In order to determine whether dormant cells residing in the lungs or in the liver could resume proliferation and regrow in the absence of T cells, an ex vivo study was performed. The lungs or liver of FVBN202 transgenic mice bearing primary mammary carcinoma who received no treatment (Ctrl), FAC chemotherapy (FAC), or FAC chemotherapy and immunotherapy (FAC/AIT) were removed and cultured in the absence of T cells, ex vivo. Adherent tumor cells were recovered from the lungs of all groups within 4–5 weeks (R-Ctrl, R-FAC, or R-FAC/AIT) and showed similar morphology as MMC (Fig. 4a). Flow cytometry analysis of FVS− viable dormant tumor cell lines that regrew ex vivo had similar characteristics by the expression of neu antigen, the epithelial cell marker EpCAM, and a predominant CD24+CD44+ fraction over CD24-CD44+ cancer stem cell-like fraction (Fig. 4b–d). Dormant tumor cells from the liver were recovered only from the FAC/AIT group 2–3 months after culture ex vivo (FAC/AIT L) and showed similar characteristics as MMC for the expression of neu, EpCAM, or predominant CD24+CD44+ fraction (Fig. S4).

### Dormant tumor cell lines are tumorigenic and establish distant tumor dormancy in the lungs and in the liver associated with the predominance of infiltrating Te and Tem phenotypes

**Fig. 5:**
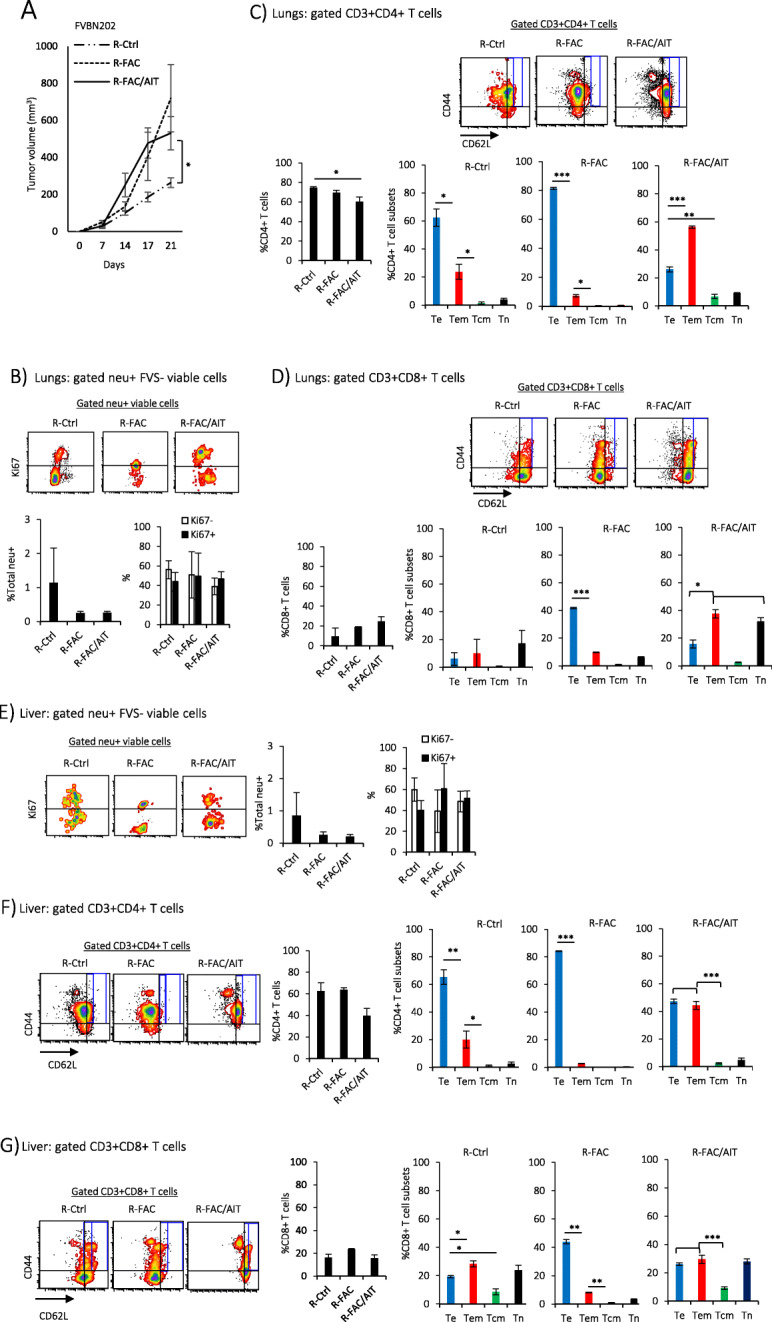
Dormant tumor cell lines establish mammary carcinoma and distant tumor dormancy in FVBN202 transgenic mice. Female FVBN202 transgenic mice (8–10 weeks old) were challenged in the mammary region with the dormant tumor cell lines (R-Ctrl, R-FAC, R-FAC/AIT, 3 million cells/mouse) which were recovered from the lungs of FVBN202 transgenic mice bearing primary mammary carcinoma. **a** Tumor growth in the mammary region was measured using a digital caliper. Percent total neu+ dormant tumor cells in the lungs (**b**) or in the liver (**e**) were detected on FVS negative (FVS−) viable cells (%total neu+ cells). Ki67 expression is shown on FVS− neu+ cells. **c**–**f** Gated FVS−CD3+ T cells were analyzed for CD4+ or CD8+ T cells in the lungs (**c**, **d**) or in the liver (**e**, **f**). Gated FVS−CD3+CD4+ or CD8+ T cells were analyzed for T effector cells (Te, CD44+CD62L−), T effector/memory cells (Tem, CD44+CD62L^low^), T central/memory cells (Tcm, CD44+CD62L^high^), or T naïve cells (Tn, CD44−CD62L+) in the lungs (**c**, **d**) or in the liver (**e**, **f**)

In order to determine if three dormant tumor cell lines recovered from the lungs and regrew ex vivo tend to migrate to the lungs when inoculated in the mammary region, FVBN202 transgenic mice were challenged with dormant tumor cell lines. As shown in Fig. 5a, all cell lines established mammary tumors in mice with the cell line from the control group showing significantly slower tumor growth. No visible tumor was detected in the lungs or in the liver of animals (Additional file 2). Flow cytometry analysis of the lungs showed the presence of neu expressing dormant tumor cells containing proportional Ki67^−^ and Ki67^low^ fractions (Fig. 5b). The lungs were primarily infiltrated with CD4+ T cells showing predominant Te > Tem in the R-Ctrl and R-FAC groups and Tem > Te in the R-FAC/AIT group (Fig. 5c). CD8+ T cells showed very low infiltration into the lungs with predominant Te > Tem and Tem/Tn > Te subsets in the R-FAC and R-FAC/AIT groups, respectively (Fig. 5d). Similar observations were made on the liver by the detection of proportional Ki67^−^ and Ki67^low^ neu expressing dormant cells (Fig. 5e). The liver was primarily infiltrated with CD4+ T cells showing predominance of Te in the R-Ctrl and R-FAC groups or the predominance of Te/Tem in the R-FAC/AIT group (Fig. 5f). CD8+ T cells showed very low infiltration into the liver with the proportional Te/Tem for the R-Ctrl and R-FAC/AIT groups or the predominance of Te cells for the R-FAC group (Fig. 5g). A dormant tumor cell line that was recovered from the liver (R-FAC/AIT L) showed a higher rate of tumorigenicity than those recovered from the lungs (R-FAC/AIT) (Fig. S5A). Flow cytometry analysis of the lungs or liver showed that R-FAC/AIT L cells established a greater number of dormant cells with proportional Ki67^−^ and Ki67^low^ fractions in the lungs (Fig. S5B) or in the liver (Fig. S5D). Analysis of infiltrating CD4+ and CD8+ T cells showed a shift from predominant Tem in the R-FAC/AIT group to predominant Te in the R-FAC/AIT L group in the lungs (Fig. S5C) and in the liver (Fig. S5E).

### Autologous tumor-sensitized T cells prevent regrowth of dormant tumor cells, ex vivo

**Fig. 6:**
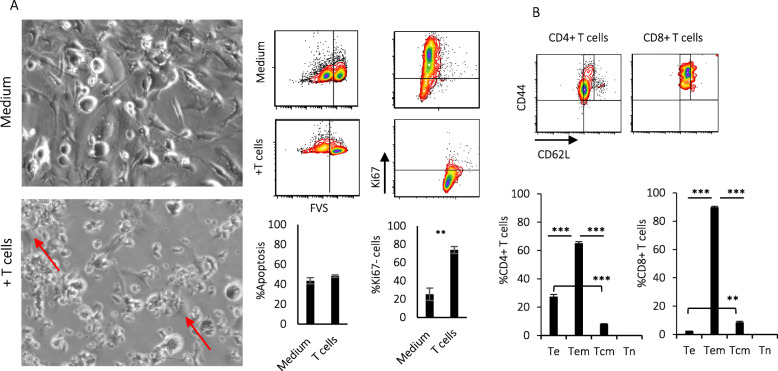
T cells predominated with Tem phenotypes inhibit the recurrence of dormant tumor cells, ex vivo. Lungs collected from FVBN202 transgenic mice who had received FAC chemotherapy were cultured alone (medium) or with tumor-reactive autologous lymphocytes (+ T cells, 4 × 10^6^ cells) 2 days after the recovery of dormant cells from the lungs in the presence of 40 unit/ml IL-2 (3 times during the culture as needed) and 20 ng/ml IL-7 (on days 5 and 9 of culture). **a** Pictures of dormant cells taken at × 20 magnification on day 12 of the co-culture. Arrows show dormant tumor cells surrounded by T cells. Tumor cell apoptosis and Ki67^−^ dormant tumor cells were analyzed on gated CD45−neu+ viable cells. **b** Gated FVS− viable T cells were analyzed for CD4+ or CD8+ T effector cells (Te, CD44+CD62L−), T effector memory cells (Tem, CD44+CD62L^low^), T central memory cells (Tcm, CD44+CD62L^high^), or T naïve cells (Tn, CD44−CD62L+)

Detection of local or distant tumor dormancy associated with the predominance of Te or Tem cells in FVBN202 transgenic mice suggests that an induction of effector T cells following tumor formation could induce and retain tumor dormancy, but cannot eliminate dormant cells. To test our hypothesis, we sought to determine whether autologous T cells from FVBN202 mice could prevent regrowth of dormant tumor cells ex vivo. Co-culture of dormant tumor cells with tumor-sensitized autologous T cells starting 2 days after the isolation of dormant cells from the lungs did not increase apoptosis in dormant cells, but resulted in the inhibition of tumor cell growth (Fig. 6a) with a predominant Ki67^−^ quiescent cells detected only in the presence of T cells (Fig. 6a). Analysis of CD4+ T cells or CD8+ T cells from the co-cultures demonstrated the predominance of Tem > Te cells or Tem cells, respectively (Fig. 6b).

### Presence of anti-tumor immune responses prior to tumor challenge results in complete protection of FVB mice from mammary carcinoma and distant tumor dormancy

**Fig. 7:**
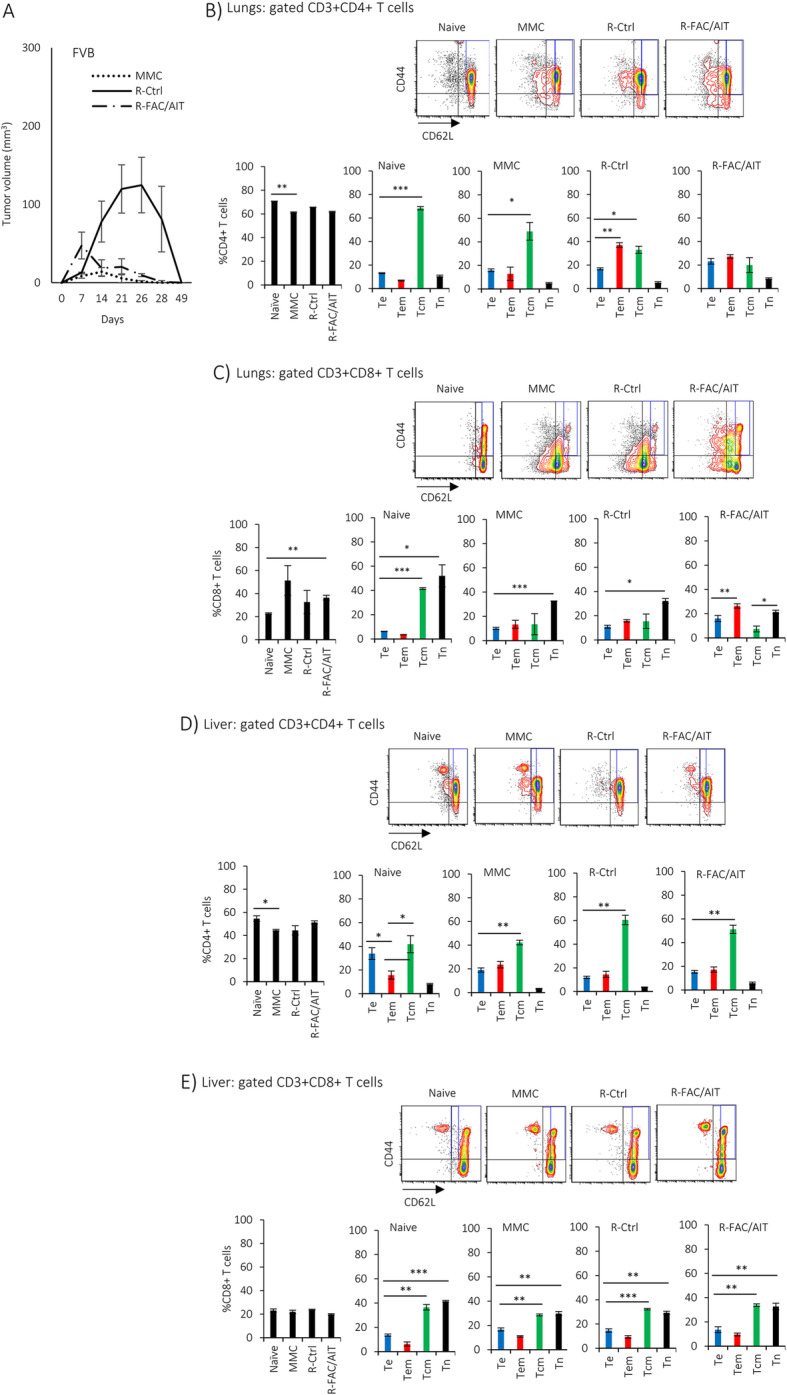
Presence of tumor-reactive immune responses prior to tumor development results in tumor rejection and prevents distant tumor dormancy in FVB mice. Female FVB mice (8–10 weeks old) were challenged with MMC, R-Ctrl, or R-FAC/AIT tumor cell lines in the mammary region (3 million cells/mouse). Naïve mice served as control. **a** Tumor growth in the mammary region was measured using a digital caliper. **b**–**e** Gated FVS−CD3+ T cells were analyzed for CD4+ or CD8+ T cells in the lungs (**b**, **c**) or in the liver (**d**, **e**). Gated FVS−CD3+CD4+ or CD8+ T cells were analyzed for T effector cells (Te, CD44+CD62L−), T effector memory cells (Tem, CD44+CD62L^low^), T central memory cells (Tcm, CD44+CD62L^high^), or T naïve cells (Tn, CD44−CD62L+) in the lungs (**b**, **c**) or in the liver (**d**, **e**)

In order to determine whether the establishment of robust anti-tumor immune response before breast cancer has become clinically detected could fully protect the host from tumor growth or distant tumor dormancy, we used FVB mice that are parental strain of FVBN202 transgenic mice, and harbor pre-existing T cell responses against a foreign antigen, rat neu protein expressed in MMC [30, 36]. Female FVB mice were challenged with MMC or the relapsed tumor cell lines R-Ctrl or R-FAC/AIT in the mammary region. Naïve FVB mice served as healthy control. All tumor cell lines were rejected by within 2–3 weeks after tumor challenge with R-Ctrl showing a slower rate of rejection within 49 days after tumor challenge (Fig. 7a). No visible tumor was detected in the lungs or in the liver, and flow cytometry analysis did not detect any neu-positive dormant tumor cells (Additional file 3). Analysis of infiltrating T cells in the lungs showed predominance of CD4+ Tcm in naïve mice and those challenged with MMC, while those challenged with R-Ctrl showed no predominance of T cell subsets (Fig. 7b). CD8+ T cells were predominated by Tn subset in all groups with the R-FAC/AIT group also showing an increased Tem subset (Fig. 7c). A dormant tumor cell line that was recovered from the liver of FVBN202 transgenic mice (R-FAC/AIT L) showed a similar rate of rejection as those recovered from the lungs (R-FAC/AIT) (Fig. S6A). Again, no neu-positive dormant cells were detected in the lungs or in the liver (Additional file 4). Analysis of T cells in the lungs and in the liver showed similar patterns for CD4+ and CD8+ T cell subsets in both groups (Fig. S6B-E).

## Discussion

The presence of tumor dormancy has been documented by showing disseminated dormant cells in the bone marrow of BALB-NeuT and MMTV-PyMT mice [37], patients with DCIS [38], and patients with breast cancer [11, 39]. Here, we demonstrated that Ki67^−^ tumor dormancy was present at the site of primary tumor in FVBN202 transgenic mice and in tumor biopsies of patients with early stage breast cancer. Presence of Ki67^−^ tumor cells in biopsies could be the trace of naturally occurring tumor dormancy, some of which could have escaped from dormancy and established primary breast cancer (Ki67^+^) and some remained in a dormant state (Ki67^−^). The Ki67^−^ dormant cells were enriched in FVBN202 transgenic mice after cancer therapies or in surgical excisions of patients with early stage breast cancer after neoadjuvant therapies. An increased proportion of Ki67^−^ dormant cells after cancer therapies is an indicative of treatment-induced tumor dormancy. Higher proportion of Ki67^−^ dormant cells than proliferating tumor cells could reduce sensitivity of tumor cells to chemotherapies which we detected in patients who did not respond to neoadjuvant therapies. In fact, chemotherapy-induced dormant cells have been reported to be in a state of senescence engulfing nondormant tumor cells to enhance their survival at the site of primary tumor during chemotherapy [40]. After cancer therapies, Ki67^+^ tumor cells became slow-cycling Ki67^low^ indolent cells. In the in vitro studies, we have reported that Ki67^low^ indolent dormant cells show a sluggish proliferation rate which was counterbalanced by spontaneous cell death so as to remain in a dormant state without being able to establish solid tumor until proliferation exceeds spontaneous cell death and result in tumor relapse [21]. Presence of local tumor dormancy as Ki67^−^ cells has also been reported in animal model of colon carcinoma [41].

In FVBN202 transgenic mice, distant tumor dormancy was also present in the lungs and in the liver very early during mammary tumor progression. The state of tumor dormancy in the lungs was not merely because of the tissue microenvironment as a systemic administration of tumor proliferating cells resulted in the establishment of mammary tumor in the lungs. Therefore, distant dormant cells may have been originated from dormant cells migrating from the primary tumor site which remained dormant in distant organs. Such disseminated dormant cells from the primary site of breast cancer have also been detected in the circulation of breast cancer survivors [42]. Such disseminated dormant cells differ from micro-metastatic tumor cells because animals did not develop lung metastasis during a follow-up after surgical removal of the primary tumor. On the other hand, migration of proliferating tumor cells to distant organs could establish metastatic cancer immediately, as shown in an experimental metastatic model by systemic administration of MMC. In other words, distant recurrence of breast cancer could be due to reawakening of disseminated dormant cells originated from primary tumor site very early during tumorigenesis. We have reported that a systemic administration of chemotherapy-induced dormant tumor cells resulted in the establishment of tumor dormancy in the lungs which eventually relapsed after a prolonged dormancy [21]. Presence of local tumor dormancy or distant tumor dormancy was associated with the predominance of infiltrating CD4+ and CD8+ Te or Tem subsets. Infiltrating CD4+ and CD8+ Te or Tem subsets might facilitate the establishment of local tumor dormancy as well as distant tumor dormancy because naïve FVBN202 transgenic mice as well as FVB mice without tumor dormancy lacked predominant Te subset. This possibility was also confirmed by demonstrating that dormant tumor cells failed to relapse in the presence of tumor-sensitized T cells but they grew in the absence of T cells, ex vivo. T cells inhibiting the growth of dormant tumor cells, ex vivo, were predominated by CD4+ and CD8+ Te or Tem subsets. This tumor inhibitory role of T cells has also been reported in other models including melanoma [43], methylcholanthrene-induced sarcoma [44], fibrosarcoma [45], and B cell lymphoma [46]. Very recently, similar observations have been made in RET transgenic mouse model of melanoma by detecting Ki67^−^ dormant tumor cells in the skin lesions, as well as in the bone marrow associated with the infiltration of Tem phenotypes [47].

Disseminated dormant tumor cells expressed the epithelial marker EpCAM and contained Ki67^−^ and Ki67^low^ fractions. This is consistent with a previous report showing that while disseminated dormant cells use a Wnt-dependent epithelial-mesenchymal transition (EMT)-like dissemination program, they retain the epithelial phenotype [48]. In the ex vivo culture, dormant tumor cells recovered faster from the lungs than from the liver despite similar patterns of T cell phenotypes being present in these organs. These data suggest additional environmental factors being present in the liver but not in the lungs inhibiting the recurrence of dormant mammary tumor cells. In fact, human liver microphysiologic system has been shown to support breast cancer dormancy [49]. This could also explain a preferential establishment of palpable mammary tumor in the lungs but not in the liver upon systemic delivery of MMC tumor cells into FVBN202 transgenic mice. Again, all dormant tumor cells that grew in culture from the lungs showed comparable levels of expression of neu or EpCAM and were predominated by the CD24+CD44+ fraction. Dormant cells that regrew ex vivo established primary tumor and distant tumor dormancy in FVBN202 transgenic mice. Unlike dormant cells established by MMC tumor cell line and predominated with Ki67^−^ fraction, the relapsed dormant cell lines established a proportionate Ki67^−^ quiescent and Ki67^low^ indolent dormant cell fractions in the lungs and in the liver of FVBN202 transgenic mice. These data suggest that tumor intrinsic factors might determine the type of tumor dormancy in the presence of tumor immune surveillance. Although similar fraction of Ki67^−^ and Ki67^low^ dormant cells were detected only in the lungs of the FAC/AIT group, this was likely because of tumor immunoediting associated with increased PD-L1 on dormant cells and decreased PD-1+ T cells. However, long-term studies are needed following surgical excision of the primary tumor to determine whether dormant cells with a proportionate Ki67^−^ quiescent and Ki67^low^ indolent fractions will relapse faster than those predominated with Ki67^−^ quiescent fraction. We have reported that Ki67^low^ indolent fraction of dormant cells is susceptible to immunoediting and subsequent tumor relapse [20, 21]. A comparative analysis of FVB and FVBN202 mice showed that pre-existing anti-tumor T cells in FVB mice could prevent distant recurrence of the disease. This suggests that administration of immunotherapy before clinical recurrences or during tumor dormancy could prevent disease recurrences in cancer survivors. We have reported that immunization of FVBN202 mice prior to tumor challenge resulted in the protection of mice from spontaneous mammary carcinoma [50]. Cancer immunotherapy could establish memory responses, thereby controlling tumor dormancy. In fact, immunotherapy is the only promising treatment for tumor dormancy because chemotherapies or radiation therapies are not effective against quiescent cells, and there is no palpable tumor to be operable [15, 19, 51]. To this end, T cells that target semi-nonself mutant neoantigens expressed on dormant cells might be highly effective because T cells from FVB mice recognizing nonself neu antigen were more potent than those from FVBN202 mice in eliminating the tumor.

## Conclusions

We demonstrated an association between the retention of tumor dormancy at distant organs and predominance of Te/Tem subsets. Te/Tem subsets inhibited regrowth of freshly isolated dormant cells from the lungs, ex vivo. Such association requires further investigation to determine whether there is a cause-effect relationship between Te/Tem cells and the retention of tumor dormancy. In addition, presence of neu-alloreactive anti-tumor T cells in FVB mice prior to tumor challenge resulted in the protection of mice from the dissemination of dormant tumor cells to distant organs. On the other hand, neu-autoreactive T cells from FVBN202 mice only prevented regrowth of dormant cells. These data suggest that immunotherapeutic targeting of semi-allogeneic mutant neoantigens during tumor dormancy might prevent distant recurrence of the disease.

## Supplementary information

Additional file 1. No metastatic tumors were detected following surgical removal of primary mammary tumor. FVBN202 mice were challenged with MMC in the mammary region and tumors were resected when they reached 800 mm3. Representative pictures of the lungs are shown. H & E stained slide is shown at 20X magnification.

Additional file 2. No visible tumor was detected in the lungs or in the liver of FVBN202 mice with primary mammary tumor. Female FVBN202 transgenic mice (8-10 weeks old) were challenged in the mammary region with the dormant tumor cell lines (R-Ctrl, R-FAC, R-FAC/AIT, 3 million cells/mouse) which were recovered from the lungs of FVBN202 transgenic mice bearing primary mammary carcinoma. Representative pictures of the lungs and liver are shown.

Additional file 3. No visible tumor or disseminated dormant tumor cells was detected in FVB mice. Female FVB mice (8-10 weeks old) were challenged with MMC, R-Ctrl or R-FAC/AIT tumor cell lines in the mammary region (3 million cells/mouse). Naïve mice served as control. Gated FVS- viable cells were analyzed for the expression of neu.

Additional file 4. No visible tumor or disseminated dormant tumor cells was detected in FVB mice following the rejection of R-FAC/AIT or R-FAC/AIT L relapsed cell lines. Female FVB mice (8-10 weeks old) were challenged with R-FAC/AIT or R-FAC/AIT L tumor cell lines in the mammary region (3 million cells/mouse). Gated FVS- viable cells were analyzed for the expression of neu.

**Additional file 5.**

## Data Availability

The datasets used and/or analyzed during the current study are available from the corresponding author on reasonable request.

## Abbreviations

ADH: Atypical ductal hyperplasia
ADR: Adriamycin
AIT: Adoptive immunotherapy
Bx: Biopsies
CYP: Cyclophosphamide
DCIS: Ductal carcinoma in situ
ER: Estrogen receptor
Ex: Surgical excisions
5-FU: Fluorouracil
FAC: 5-FU + ADR + CYP
HER-2: Human epidermal growth factor receptor 2
MMC: Mouse mammary carcinoma
MMTV: Mouse mammary tumor virus promoter
PR: Progesterone receptor
