# Supplementary figures and images for: Local and distant tumor dormancy during early stage breast cancer are associated with the predominance of infiltrating T effector subsets
Source: Breast Cancer Res. 2020 Oct 28;22:116. doi: 10.1186/s13058-020-01357-9 (PMC7594332; doi:10.1186/s13058-020-01357-9)

## Slide 1
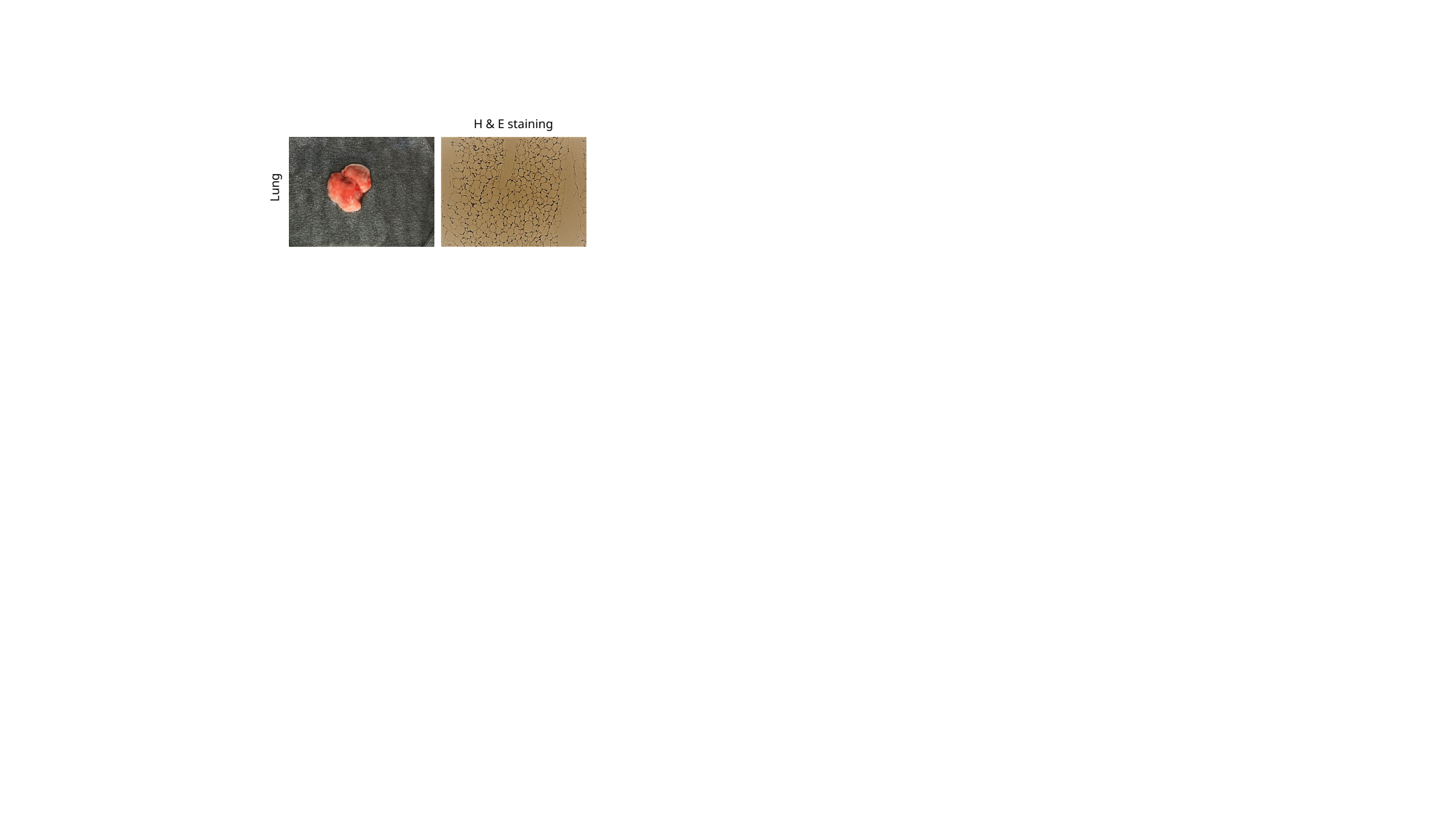

H & E staining
Lung

Supplement: Supplementary file 1 — No metastatic tumors were detected following surgical removal of primary mammary tumor. FVBN202 mice were challenged with MMC in the mammary region and tumors were resected when they reached 800 mm3. Representative pictures of the lungs are shown. H & E stained slide is shown at 20X magnification. [file 13058_2020_1357_MOESM1_ESM.pptx]

## Slide 1
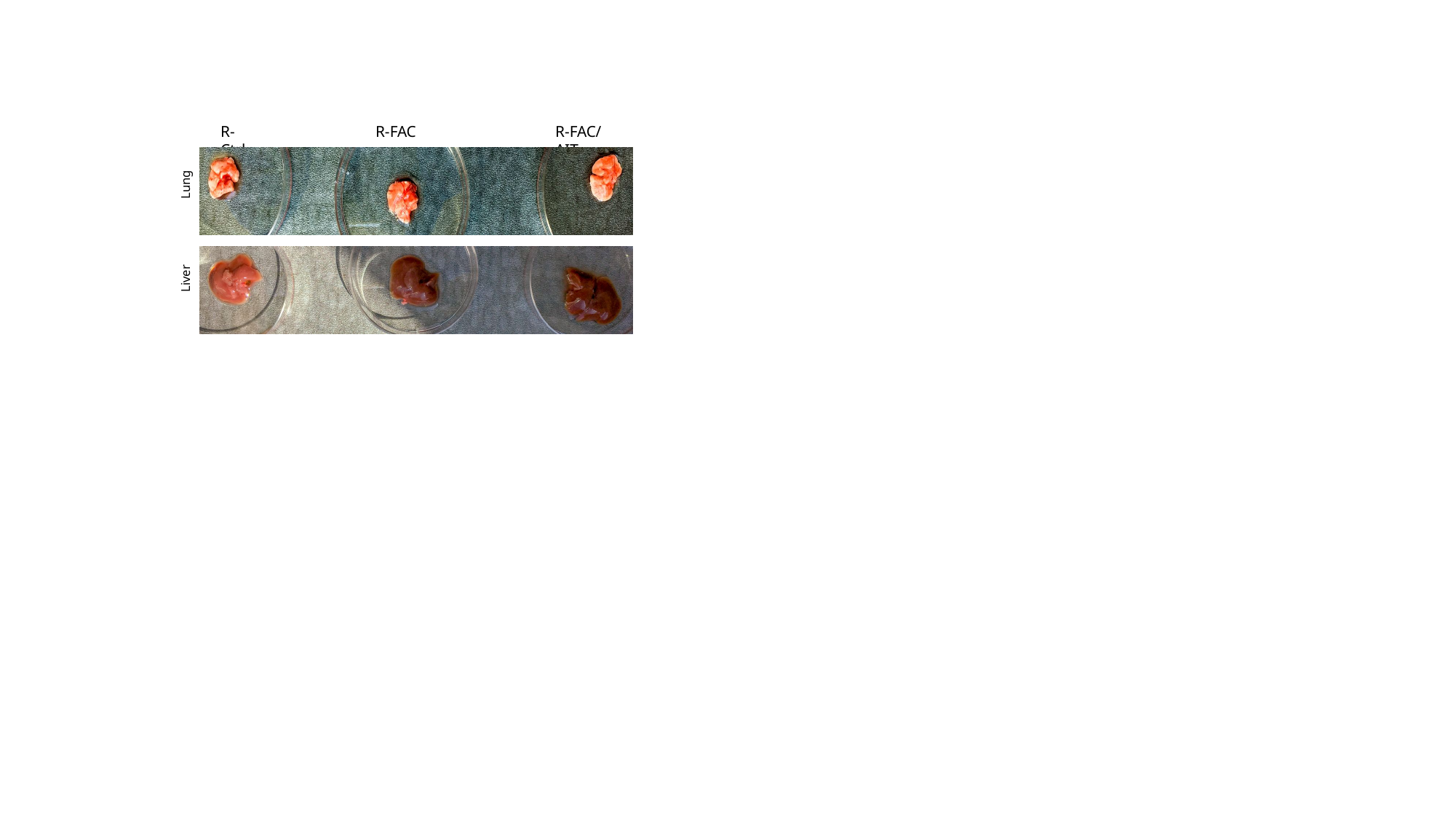

R-Ctrl
R-FAC
R-FAC/AIT
Lung
Liver

Supplement: Supplementary file 2 — No visible tumor was detected in the lungs or in the liver of FVBN202 mice with primary mammary tumor. Female FVBN202 transgenic mice (8-10 weeks old) were challenged in the mammary region with the dormant tumor cell lines (R-Ctrl, R-FAC, R-FAC/AIT, 3 million cells/mouse) which were recovered from the lungs of FVBN202 transgenic mice bearing primary mammary carcinoma. Representative pictures of the lungs and liver are shown. [file 13058_2020_1357_MOESM2_ESM.pptx]

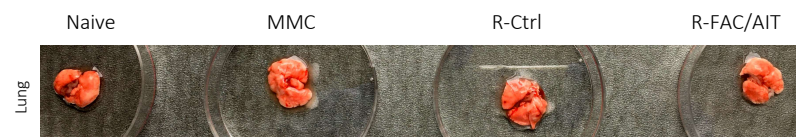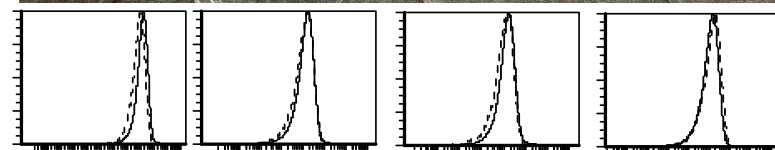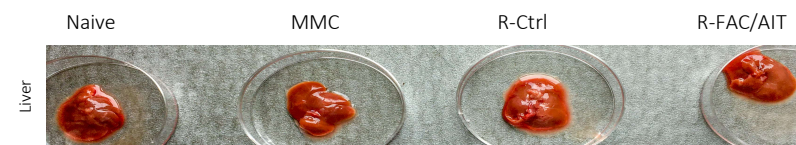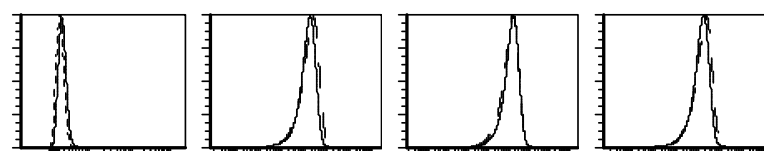

Supplement: Supplementary file 3 — No visible tumor or disseminated dormant tumor cells was detected in FVB mice. Female FVB mice (8-10 weeks old) were challenged with MMC, R-Ctrl or R-FAC/AIT tumor cell lines in the mammary region (3 million cells/mouse). Naïve mice served as control. Gated FVS- viable cells were analyzed for the expression of neu. [file 13058_2020_1357_MOESM3_ESM.pdf]

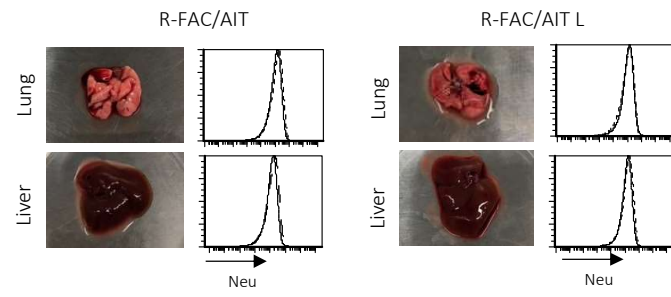

Supplement: Supplementary file 4 — No visible tumor or disseminated dormant tumor cells was detected in FVB mice following the rejection of R-FAC/AIT or R-FAC/AIT L relapsed cell lines. Female FVB mice (8-10 weeks old) were challenged with R-FAC/AIT or R-FAC/AIT L tumor cell lines in the mammary region (3 million cells/mouse). Gated FVS- viable cells were analyzed for the expression of neu. [file 13058_2020_1357_MOESM4_ESM.pdf]
