# Supplementary material for: Local and distant tumor dormancy during early stage breast cancer are associated with the predominance of infiltrating T effector subsets
Source: Breast Cancer Res. 2020 Oct 28;22:116. doi: 10.1186/s13058-020-01357-9 (PMC7594332; doi:10.1186/s13058-020-01357-9)
Supplement: Supplementary file 5 — Additional file 5. [file 13058_2020_1357_MOESM5_ESM.pptx]

## Slide 1
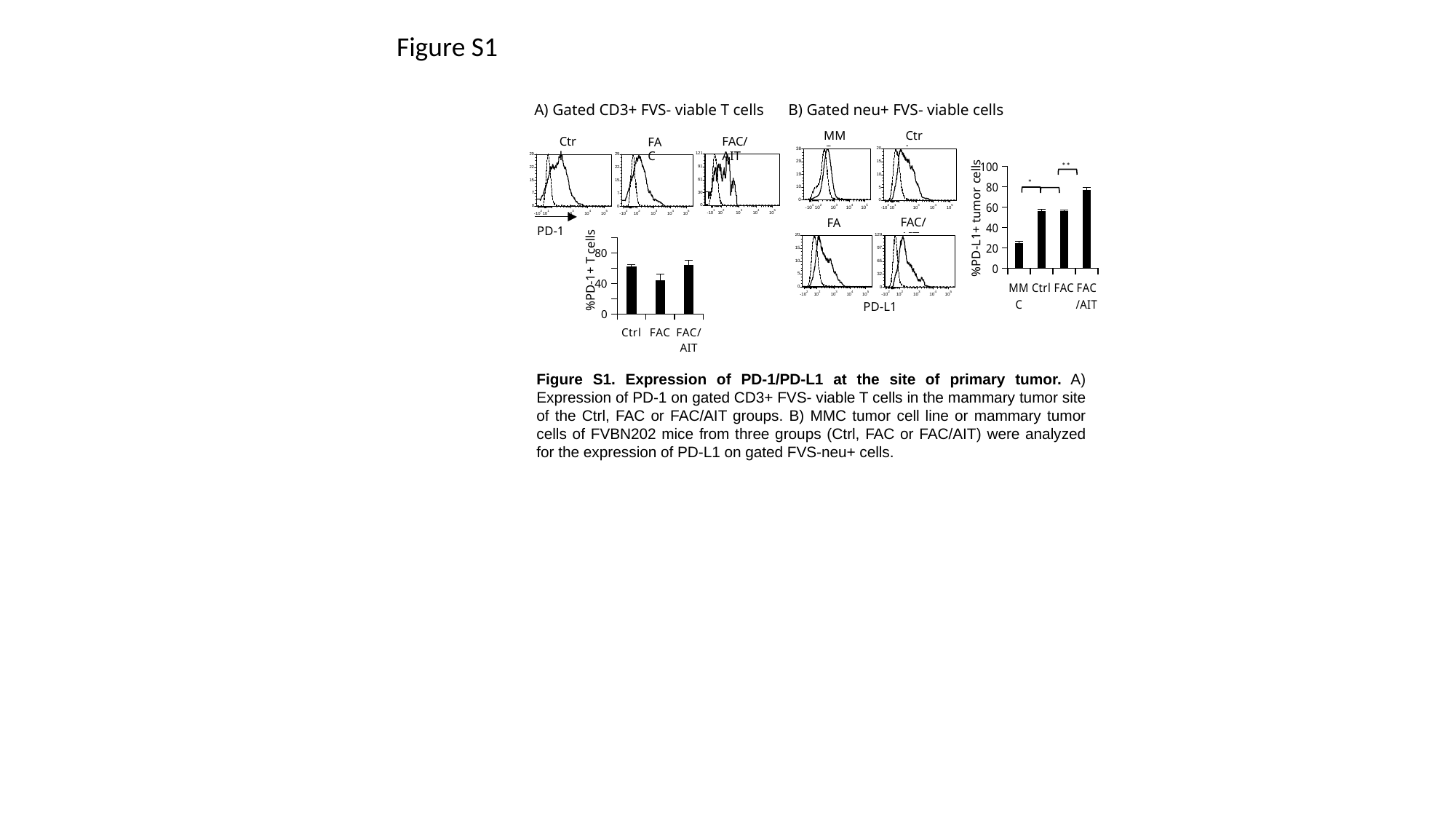

Figure S1
A) Gated CD3+ FVS- viable T cells
B) Gated neu+ FVS- viable cells
MMC
Ctrl
FAC/AIT
Ctrl
FAC
### Chart
| Category | |
|---|---|
| MMC | 24.12333333333328 |
| Ctrl | 55.485 |
| FAC | 55.39666666666658 |
| FAC/AIT | 76.21 |
**
*
%PD-L1+ tumor cells
FAC/AIT
FAC
PD-1
### Chart
| Category | |
|---|---|
| Ctrl | 61.59 |
| FAC | 43.97000000000001 |
| FAC/AIT | 63.13666666666659 |
%PD-1+ T cells
PD-L1
Figure S1. Expression of PD-1/PD-L1 at the site of primary tumor. A) Expression of PD-1 on gated CD3+ FVS- viable T cells in the mammary tumor site of the Ctrl, FAC or FAC/AIT groups. B) MMC tumor cell line or mammary tumor cells of FVBN202 mice from three groups (Ctrl, FAC or FAC/AIT) were analyzed for the expression of PD-L1 on gated FVS-neu+ cells.

## Slide 2
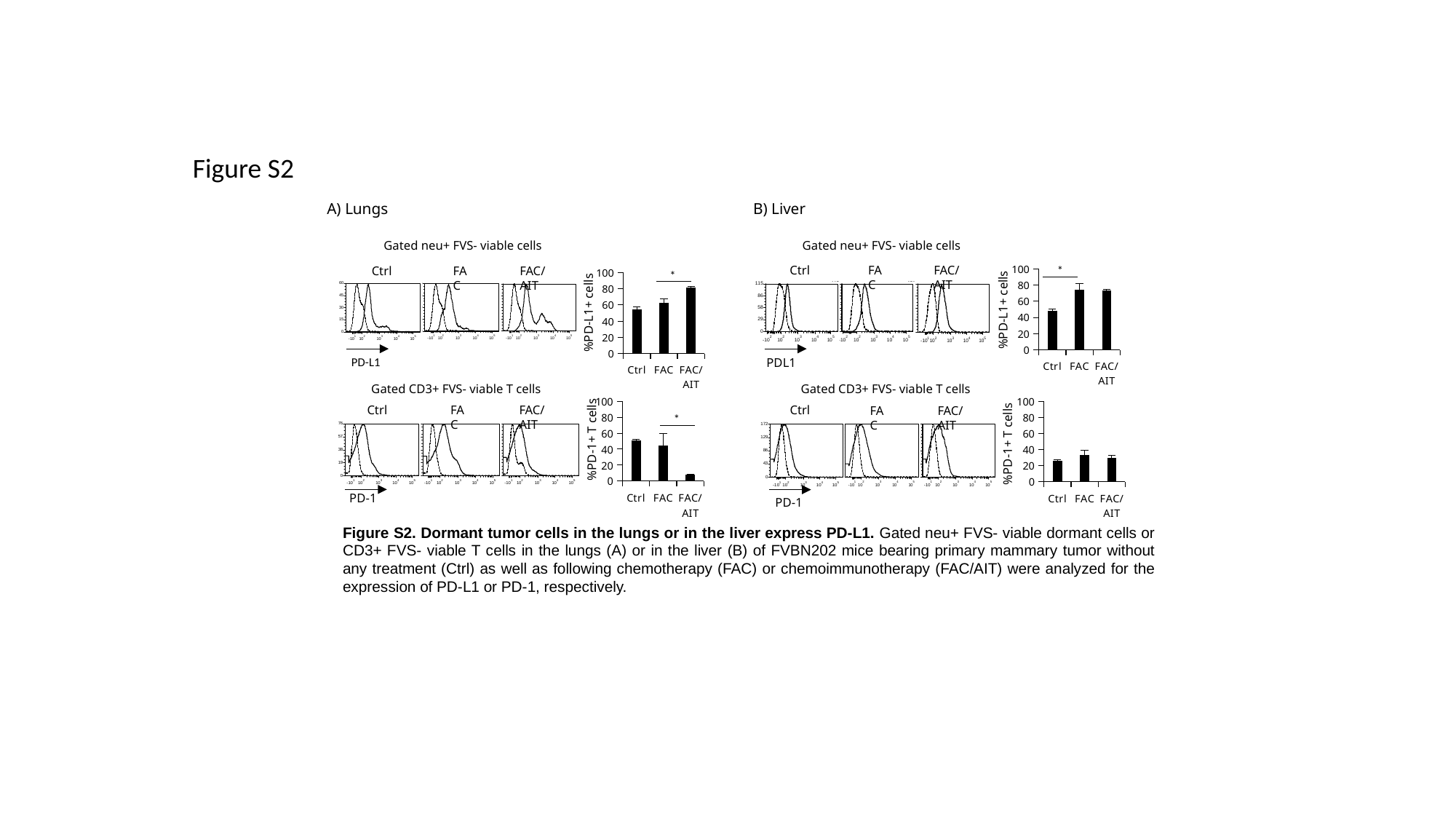

Figure S2
A) Lungs
B) Liver
Gated neu+ FVS- viable cells
Gated neu+ FVS- viable cells
### Chart
| Category | |
|---|---|
| Ctrl | 47.84 |
| FAC | 73.80333333333327 |
| FAC/AIT | 72.73333333333328 |*
Ctrl
FAC
FAC/AIT
Ctrl
FAC
FAC/AIT
### Chart
| Category | |
|---|---|
| Ctrl | 54.22 |
| FAC | 62.49666666666659 |
| FAC/AIT | 81.2466666666667 |*
%PD-L1+ cells
%PD-L1+ cells
PD-L1
PDL1
Gated CD3+ FVS- viable T cells
Gated CD3+ FVS- viable T cells
### Chart
| Category | |
|---|---|
| Ctrl | 50.28 |
| FAC | 44.17333333333334 |
| FAC/AIT | 7.8 |
### Chart
| Category | |
|---|---|
| Ctrl | 25.68 |
| FAC | 32.90666666666656 |
| FAC/AIT | 28.77 |Ctrl
Ctrl
FAC
FAC/AIT
FAC
FAC/AIT
*
%PD-1+ T cells
%PD-1+ T cells
PD-1
PD-1
Figure S2. Dormant tumor cells in the lungs or in the liver express PD-L1. Gated neu+ FVS- viable dormant cells or CD3+ FVS- viable T cells in the lungs (A) or in the liver (B) of FVBN202 mice bearing primary mammary tumor without any treatment (Ctrl) as well as following chemotherapy (FAC) or chemoimmunotherapy (FAC/AIT) were analyzed for the expression of PD-L1 or PD-1, respectively.

## Slide 3
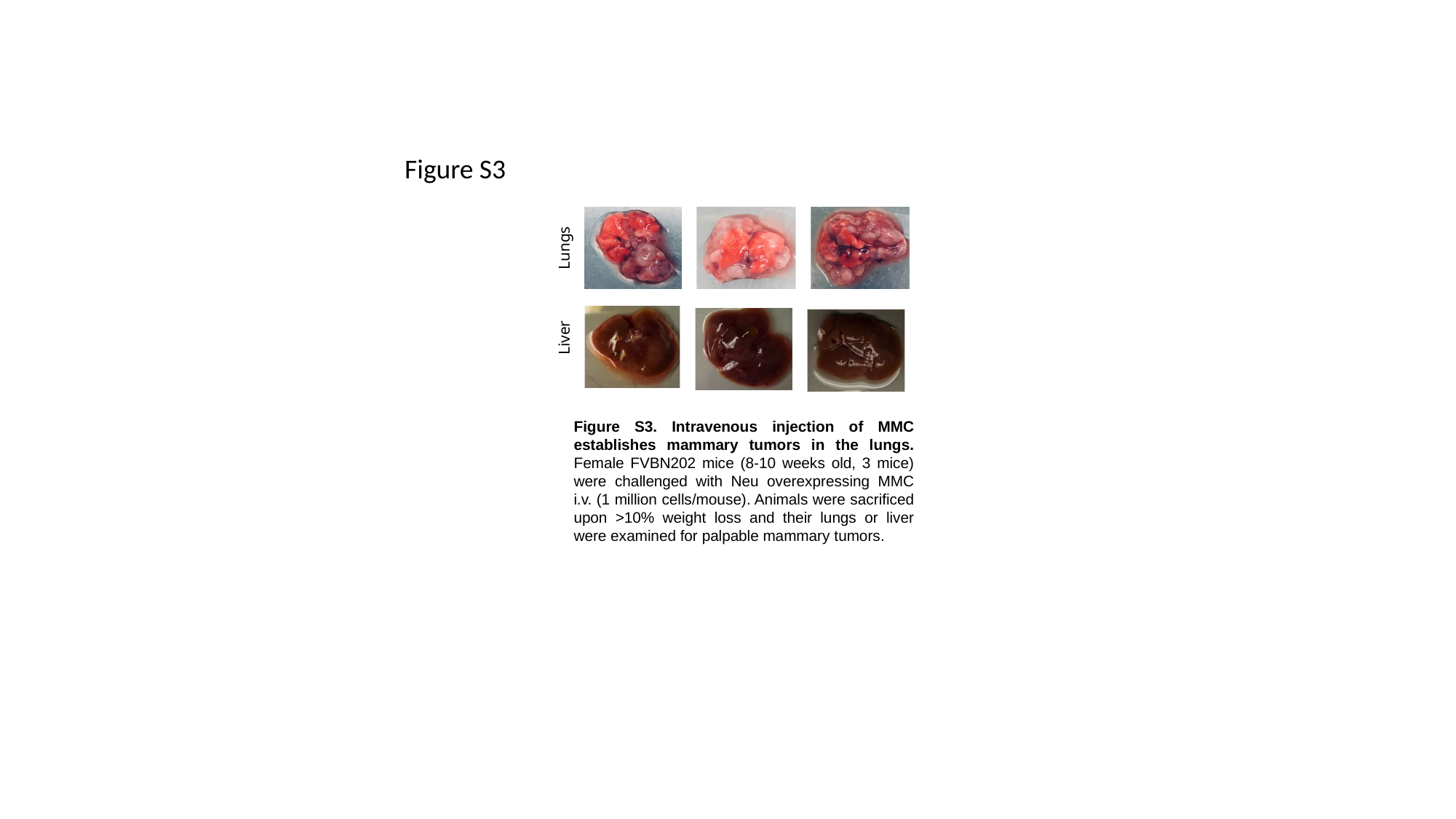

Figure S3
Lungs
Liver
Figure S3. Intravenous injection of MMC establishes mammary tumors in the lungs. Female FVBN202 mice (8-10 weeks old, 3 mice) were challenged with Neu overexpressing MMC i.v. (1 million cells/mouse). Animals were sacrificed upon >10% weight loss and their lungs or liver were examined for palpable mammary tumors.

## Slide 4
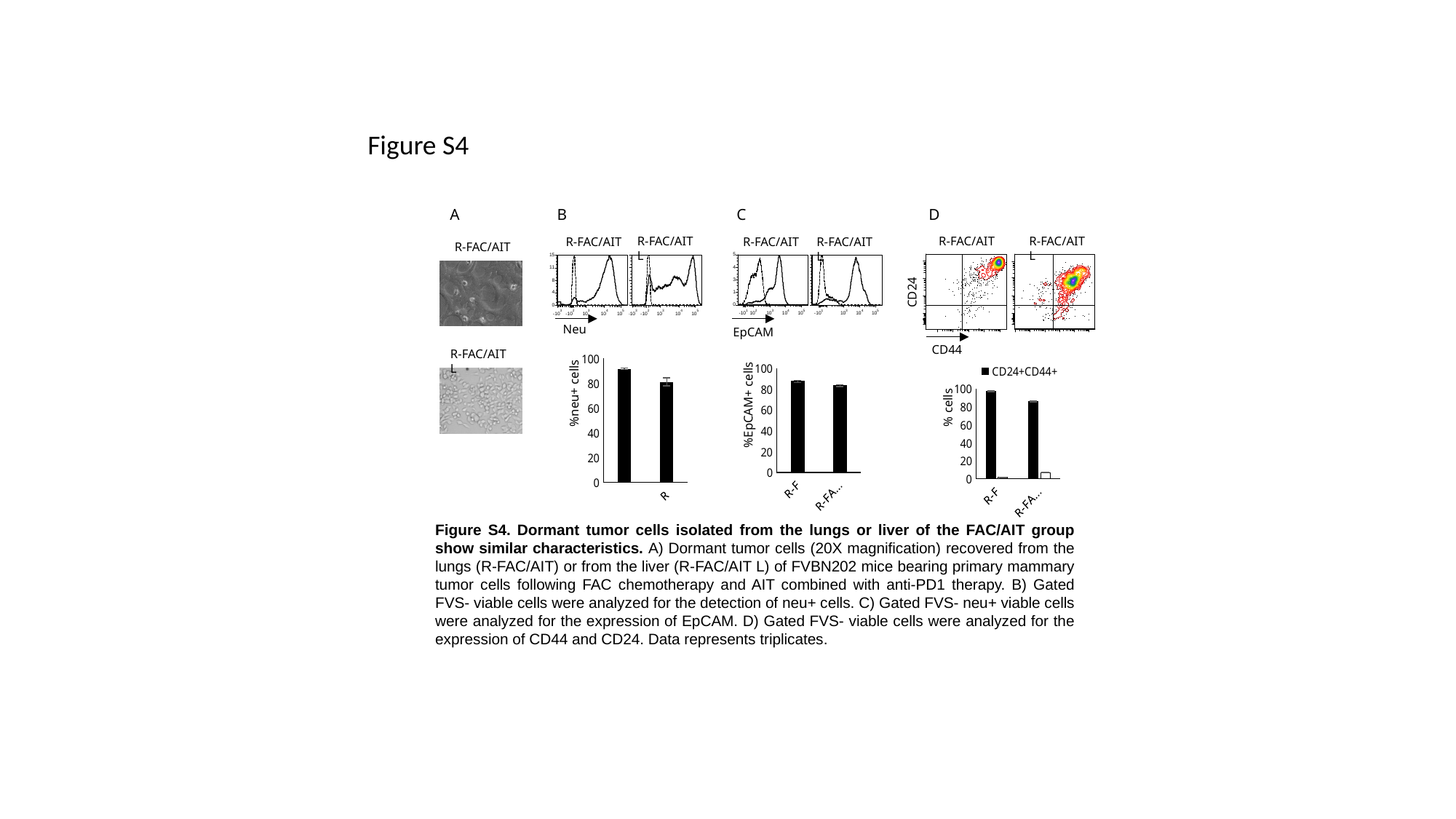

Figure S4
A
B
C
D
R-FAC/AIT L
R-FAC/AIT L
R-FAC/AIT
R-FAC/AIT
R-FAC/AIT
R-FAC/AIT L
R-FAC/AIT
CD24
Neu
EpCAM
CD44
### Chart
| Category | |
|---|---|
| R-FAC/AIT | 91.73333333333328 |
| R-FAC/AIT L | 81.14666666666666 |R-FAC/AIT L
### Chart
| Category | EpCAM |
|---|---|
| R-FAC/AIT | 87.17999999999998 |
| R-FAC/AIT L | 83.06666666666666 |
### Chart
| Category | CD24+CD44+ | CD24-CD44+ |
|---|---|---|
| R-FAC/AIT | 96.75666666666667 | 1.346666666666667 |
| R-FAC/AIT L | 85.60333333333321 | 6.88 |
%neu+ cells
%EpCAM+ cells
% cells
Figure S4. Dormant tumor cells isolated from the lungs or liver of the FAC/AIT group show similar characteristics. A) Dormant tumor cells (20X magnification) recovered from the lungs (R-FAC/AIT) or from the liver (R-FAC/AIT L) of FVBN202 mice bearing primary mammary tumor cells following FAC chemotherapy and AIT combined with anti-PD1 therapy. B) Gated FVS- viable cells were analyzed for the detection of neu+ cells. C) Gated FVS- neu+ viable cells were analyzed for the expression of EpCAM. D) Gated FVS- viable cells were analyzed for the expression of CD44 and CD24. Data represents triplicates.

## Slide 5
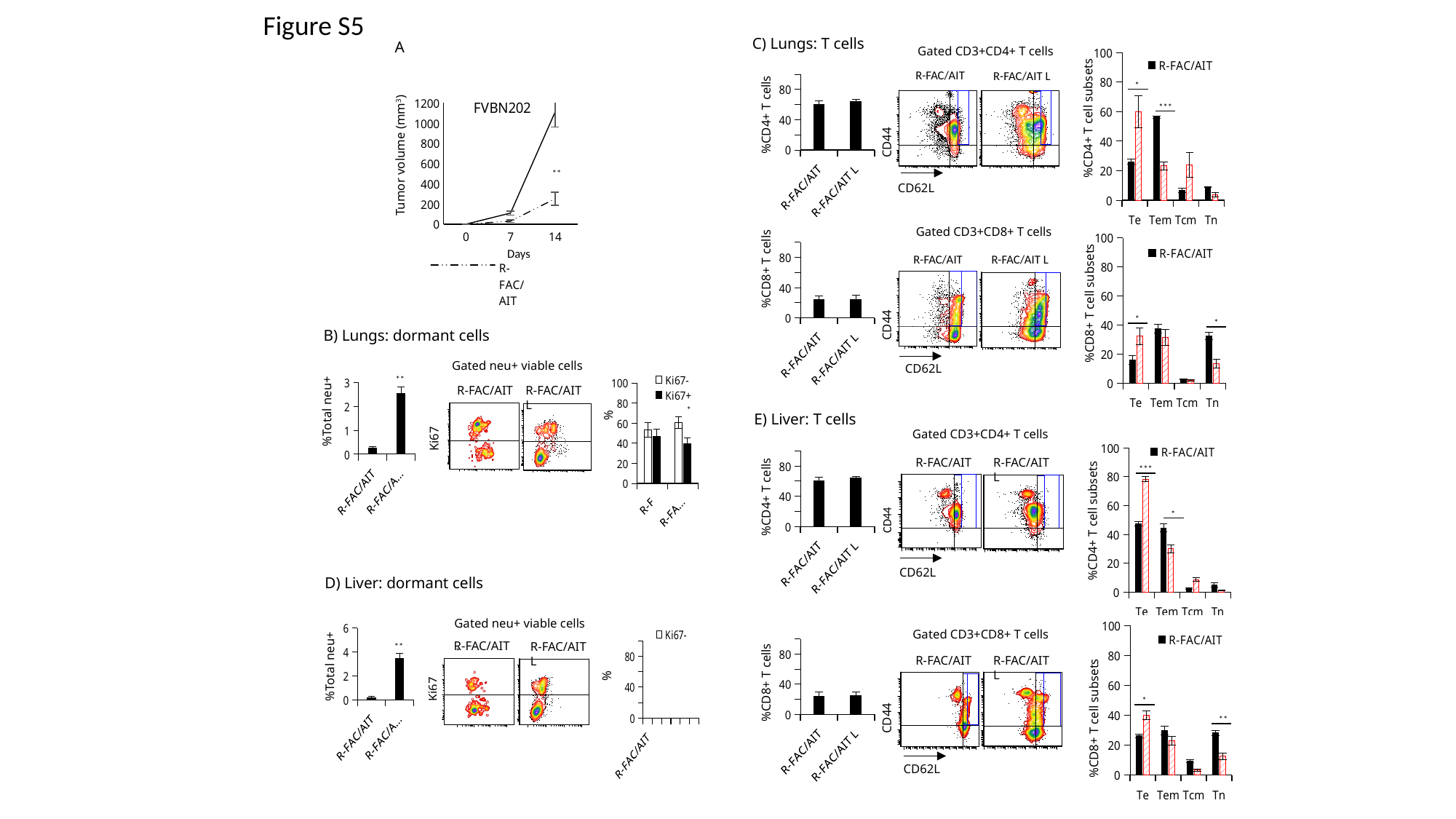

Figure S5
C) Lungs: T cells
A
Gated CD3+CD4+ T cells
### Chart
| Category | R-FAC/AIT | R-FAC/AIT L |
|---|---|---|
| Te | 26.01 | 60.15 |
| Tem | 56.23333333333333 | 23.38666666666667 |
| Tcm | 6.656666666666666 | 23.93666666666667 |
| Tn | 8.89666666666667 | 3.936666666666667 |
### Chart
| Category | |
|---|---|
| R-FAC/AIT | 60.50333333333333 |
| R-FAC/AIT L | 64.52 |R-FAC/AIT
R-FAC/AIT L
*
### Chart: FVBN202
| Category | R-FAC/AIT | R-FAC/AIT L |
|---|---|---|
| 0 | 0.0 | 0.0 |
| 7 | 29.33333333333328 | 109.3333333333333 |
| 14 | 250.6666666666666 | 1107.333333333333 |***
%CD4+ T cells
%CD4+ T cell subsets
CD44
Tumor volume (mm3)
**
CD62L
Gated CD3+CD8+ T cells
### Chart
| Category | |
|---|---|
| R-FAC/AIT | 24.81333333333328 |
| R-FAC/AIT L | 24.98333333333325 |
### Chart
| Category | R-FAC/AIT | R-FAC/AIT L |
|---|---|---|
| Te | 15.81 | 32.3 |
| Tem | 37.57666666666661 | 31.28666666666666 |
| Tcm | 2.48 | 1.866666666666666 |
| Tn | 32.25666666666661 | 13.28666666666667 |Days
R-FAC/AIT
R-FAC/AIT L
%CD8+ T cells
%CD8+ T cell subsets
*
*
CD44
B) Lungs: dormant cells
Gated neu+ viable cells
CD62L
### Chart
| Category | |
|---|---|
| R-FAC/AIT | 0.26 |
| R-FAC/AIT L | 2.54 |
### Chart
| Category | Ki67- | Ki67+ |
|---|---|---|
| R-FAC/AIT | 53.26 | 46.74 |
| R-FAC/AIT L | 60.52333333333333 | 39.47666666666651 |**
R-FAC/AIT
R-FAC/AIT L
*
%Total neu+
%
E) Liver: T cells
Gated CD3+CD4+ T cells
Ki67
### Chart
| Category | R-FAC/AIT | R-FAC/AIT L |
|---|---|---|
| Te | 47.31 | 78.31333333333329 |
| Tem | 44.3766666666666 | 30.02 |
| Tcm | 2.306666666666666 | 8.563333333333334 |
| Tn | 4.693333333333333 | 1.256666666666667 |
### Chart
| Category | |
|---|---|
| R-FAC/AIT | 60.50333333333333 |
| R-FAC/AIT L | 64.52 |R-FAC/AIT
R-FAC/AIT L
***
%CD4+ T cells
*
CD44
%CD4+ T cell subsets
CD62L
D) Liver: dormant cells
Gated neu+ viable cells
### Chart
| Category | |
|---|---|
| R-FAC/AIT | 0.21 |
| R-FAC/AIT L | 3.503333333333334 |
### Chart
| Category | R-FAC/AIT | R-FAC/AIT L |
|---|---|---|
| Te | 26.09666666666667 | 39.9166666666666 |
| Tem | 29.5533333333333 | 22.97 |
| Tcm | 9.26 | 3.27 |
| Tn | 28.10666666666667 | 12.57333333333333 |
### Chart
| Category | Ki67- | Ki67+ |
|---|---|---|
| R-FAC/AIT | 48.53333333333333 | 51.46666666666653 |
| R-FAC/AIT L | 62.23 | 37.77 |
### Chart
| Category | |
|---|---|
| R-FAC/AIT | 24.81333333333328 |
| R-FAC/AIT L | 24.98333333333325 |Gated CD3+CD8+ T cells
R-FAC/AIT
R-FAC/AIT L
**
*
R-FAC/AIT
R-FAC/AIT L
%Total neu+
%
%CD8+ T cells
Ki67
*
CD44
%CD8+ T cell subsets
**
CD62L

## Slide 6
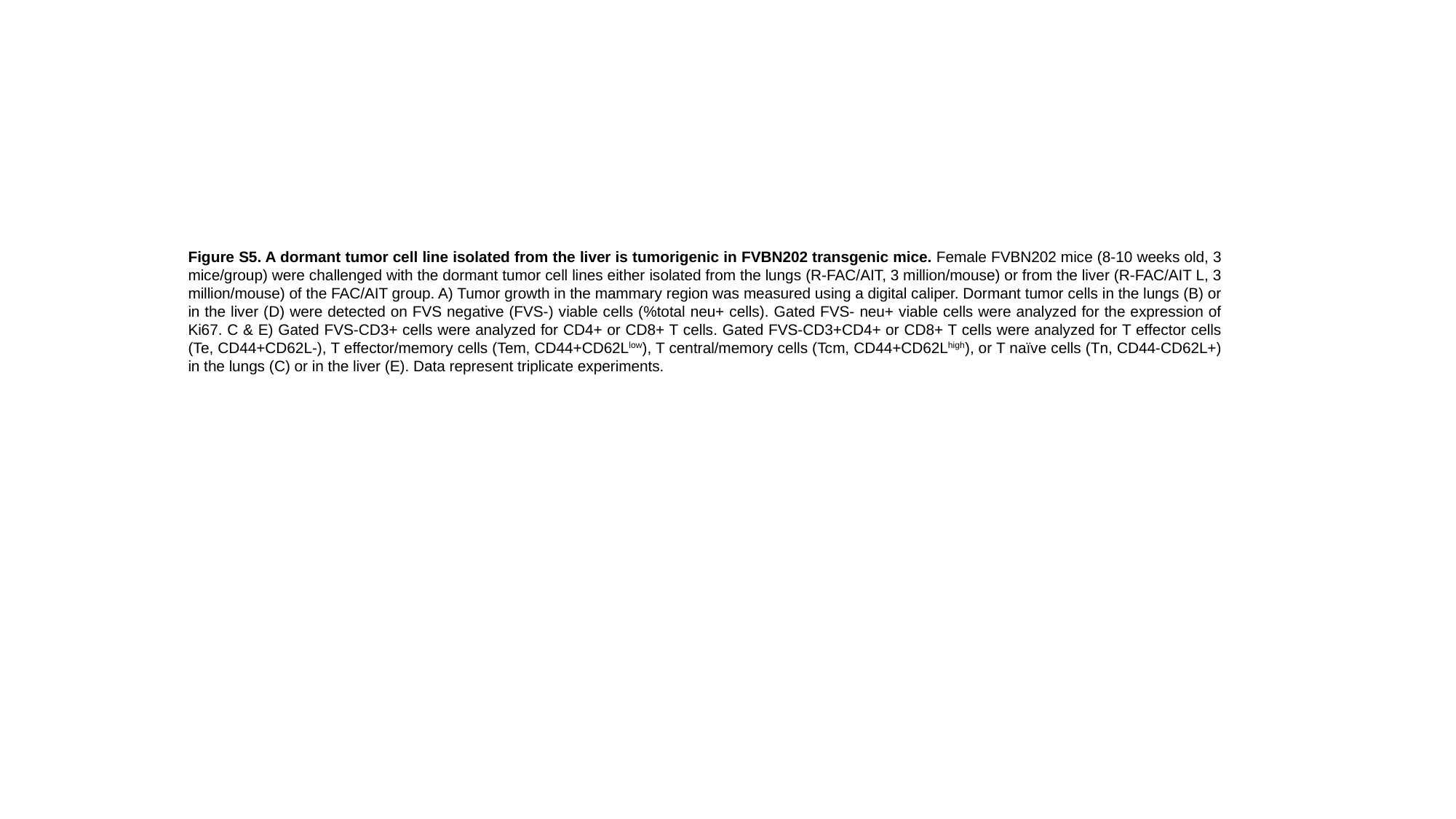

Figure S5. A dormant tumor cell line isolated from the liver is tumorigenic in FVBN202 transgenic mice. Female FVBN202 mice (8-10 weeks old, 3 mice/group) were challenged with the dormant tumor cell lines either isolated from the lungs (R-FAC/AIT, 3 million/mouse) or from the liver (R-FAC/AIT L, 3 million/mouse) of the FAC/AIT group. A) Tumor growth in the mammary region was measured using a digital caliper. Dormant tumor cells in the lungs (B) or in the liver (D) were detected on FVS negative (FVS-) viable cells (%total neu+ cells). Gated FVS- neu+ viable cells were analyzed for the expression of Ki67. C & E) Gated FVS-CD3+ cells were analyzed for CD4+ or CD8+ T cells. Gated FVS-CD3+CD4+ or CD8+ T cells were analyzed for T effector cells (Te, CD44+CD62L-), T effector/memory cells (Tem, CD44+CD62Llow), T central/memory cells (Tcm, CD44+CD62Lhigh), or T naïve cells (Tn, CD44-CD62L+) in the lungs (C) or in the liver (E). Data represent triplicate experiments.

## Slide 7
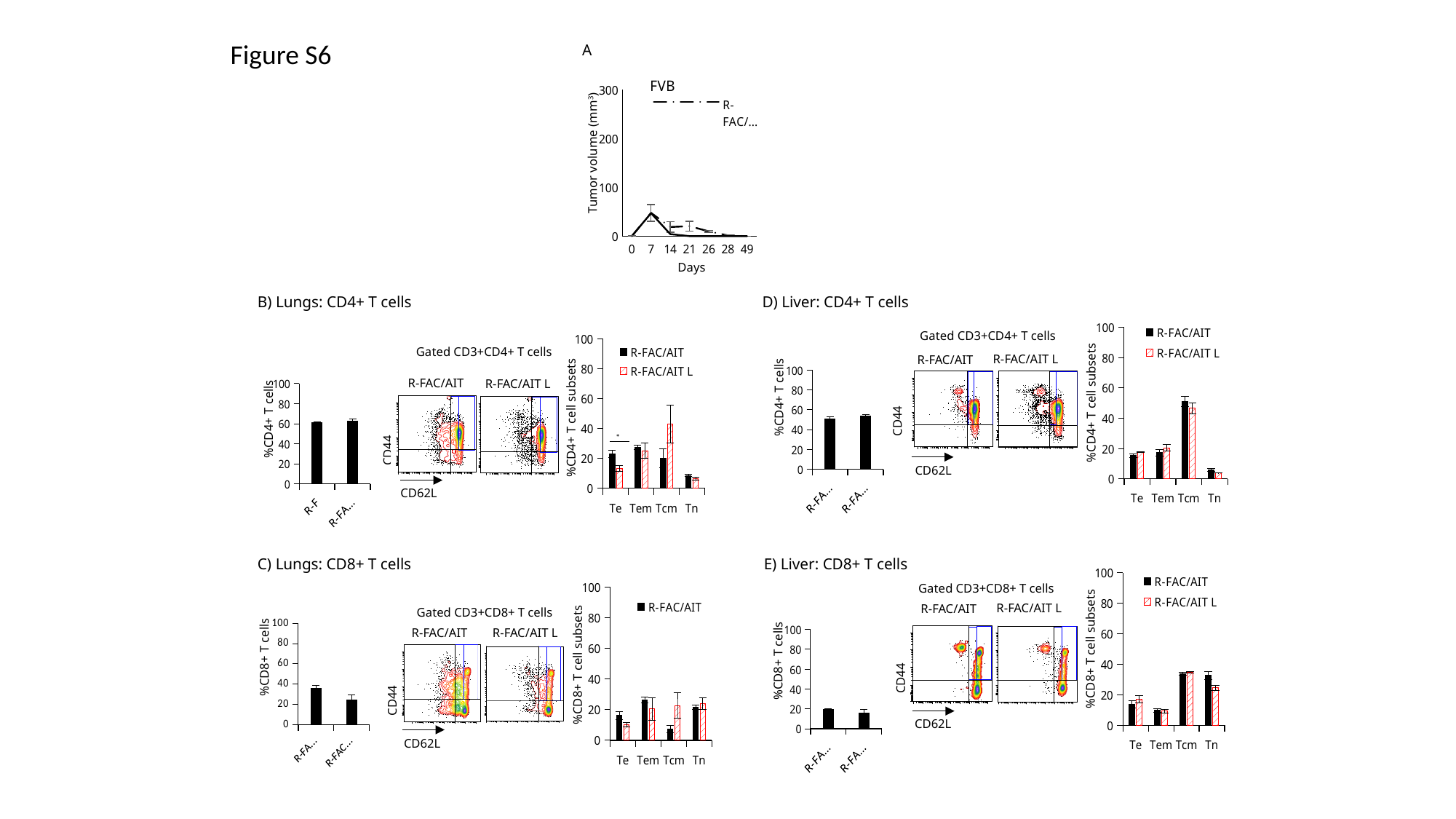

Figure S6
A
### Chart: FVB
| Category | R-FAC/AIT | R-FAC/AIT L |
|---|---|---|
| 0 | 0.0 | 0.0 |
| 7 | 47.53333333333333 | 47.0 |
| 14 | 18.66666666666667 | 4.0 |
| 21 | 20.33333333333328 | 0.0 |
| 26 | 9.700000000000001 | 0.0 |
| 28 | 1.333333333333333 | 0.0 |
| 49 | 0.0 | 0.0 |Tumor volume (mm3)
Days
B) Lungs: CD4+ T cells
D) Liver: CD4+ T cells
### Chart
| Category | R-FAC/AIT | R-FAC/AIT L |
|---|---|---|
| Te | 15.28666666666667 | 17.74333333333331 |
| Tem | 17.2433333333333 | 20.45 |
| Tcm | 51.17333333333334 | 46.56666666666663 |
| Tn | 5.819999999999998 | 3.883333333333334 |Gated CD3+CD4+ T cells
### Chart
| Category | R-FAC/AIT | R-FAC/AIT L |
|---|---|---|
| Te | 23.1733333333333 | 13.17333333333333 |
| Tem | 27.28666666666666 | 25.18333333333331 |
| Tcm | 19.98999999999998 | 42.94333333333333 |
| Tn | 8.480000000000002 | 6.49 |Gated CD3+CD4+ T cells
R-FAC/AIT L
R-FAC/AIT
### Chart
| Category | |
|---|---|
| R-FAC/AIT | 51.25666666666653 |
| R-FAC/AIT L | 53.68333333333334 |
### Chart
| Category | |
|---|---|
| R-FAC/AIT | 61.70000000000001 |
| R-FAC/AIT L | 63.12666666666657 |
R-FAC/AIT
R-FAC/AIT L
%CD4+ T cells
%CD4+ T cell subsets
%CD4+ T cell subsets
%CD4+ T cells
CD44
*
CD44
CD62L
CD62L
C) Lungs: CD8+ T cells
E) Liver: CD8+ T cells
### Chart
| Category | R-FAC/AIT | R-FAC/AIT L |
|---|---|---|
| Te | 13.61333333333333 | 17.19 |
| Tem | 9.723333333333331 | 9.283333333333333 |
| Tcm | 33.78333333333333 | 34.73666666666663 |
| Tn | 32.71333333333333 | 24.65333333333331 |
### Chart
| Category | R-FAC/AIT | R-FAC/AIT L |
|---|---|---|
| Te | 16.0633333333333 | 10.29333333333333 |
| Tem | 26.32666666666666 | 20.3433333333333 |
| Tcm | 7.413333333333333 | 22.62666666666667 |
| Tn | 21.45666666666667 | 24.06 |Gated CD3+CD8+ T cells
R-FAC/AIT L
R-FAC/AIT
Gated CD3+CD8+ T cells
### Chart
| Category | |
|---|---|
| R-FAC/AIT | 36.52333333333333 |
| R-FAC/AIT L | 25.00666666666667 |
### Chart
| Category | |
|---|---|
| R-FAC/AIT | 19.81 |
| R-FAC/AIT L | 16.15333333333328 |R-FAC/AIT
R-FAC/AIT L
%CD8+ T cell subsets
%CD8+ T cells
%CD8+ T cells
%CD8+ T cell subsets
CD44
CD44
CD62L
CD62L

## Slide 8
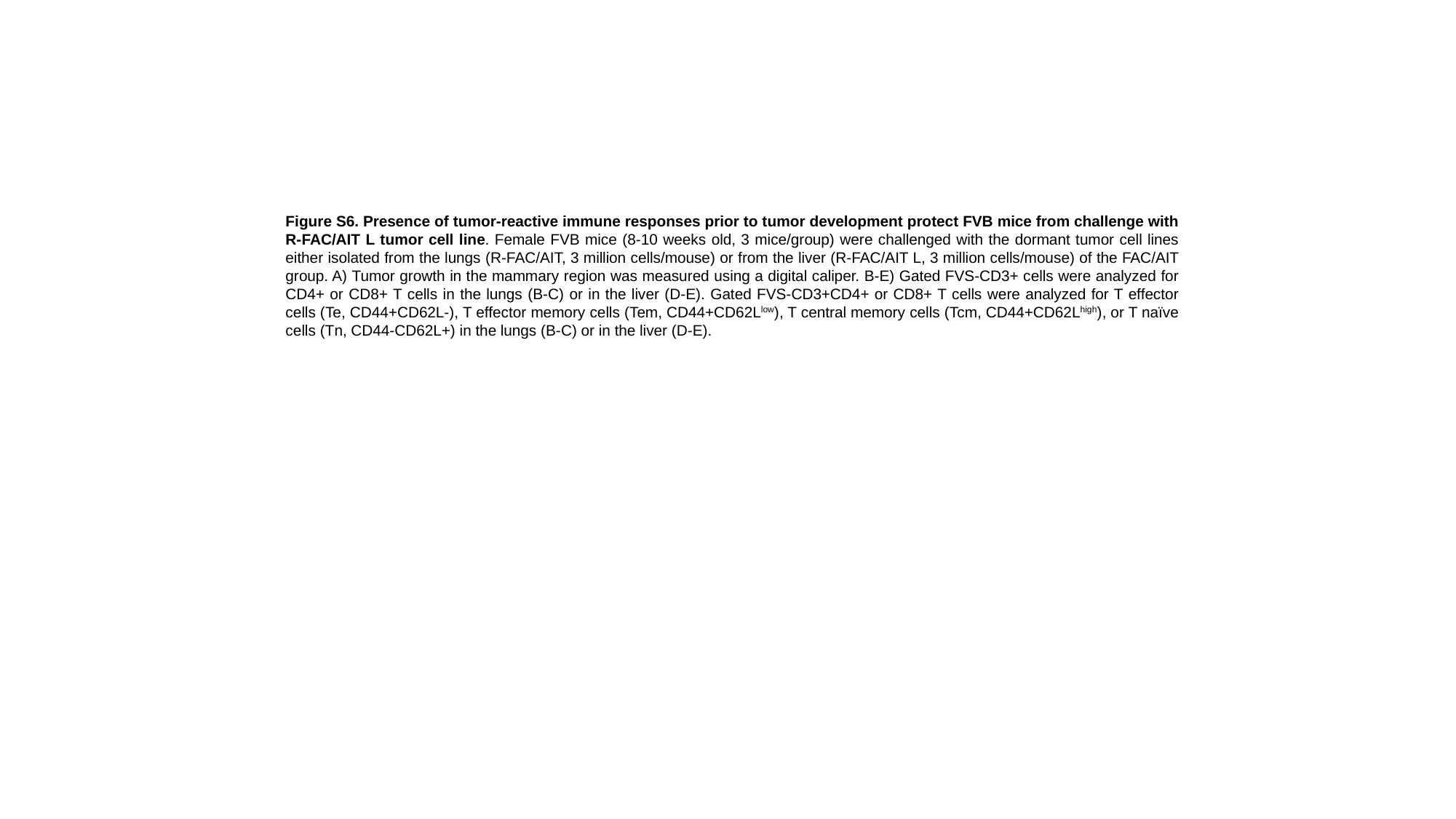

Figure S6. Presence of tumor-reactive immune responses prior to tumor development protect FVB mice from challenge with R-FAC/AIT L tumor cell line. Female FVB mice (8-10 weeks old, 3 mice/group) were challenged with the dormant tumor cell lines either isolated from the lungs (R-FAC/AIT, 3 million cells/mouse) or from the liver (R-FAC/AIT L, 3 million cells/mouse) of the FAC/AIT group. A) Tumor growth in the mammary region was measured using a digital caliper. B-E) Gated FVS-CD3+ cells were analyzed for CD4+ or CD8+ T cells in the lungs (B-C) or in the liver (D-E). Gated FVS-CD3+CD4+ or CD8+ T cells were analyzed for T effector cells (Te, CD44+CD62L-), T effector memory cells (Tem, CD44+CD62Llow), T central memory cells (Tcm, CD44+CD62Lhigh), or T naïve cells (Tn, CD44-CD62L+) in the lungs (B-C) or in the liver (D-E).
